# Engineering *Nicotiana benthamiana* for chrysoeriol production using synthetic biology approaches

**DOI:** 10.3389/fpls.2024.1458916

**Published:** 2024-12-17

**Authors:** Saet Buyl Lee, Sung-eun Lee, Hyo Lee, Ji-Su Kim, Hyoseon Choi, Sichul Lee, Beom-Gi Kim

**Affiliations:** Metabolic Engineering Division, National Institute of Agricultural Science, Rural Development Administration, JeonJu, Republic of Korea

**Keywords:** chrysoeriol, co-expression, flavonoid, gene combination, Nicotiana benthamiana, reconstruction, synthetic biology, transient expression

## Abstract

Flavonoids are prevalent plant secondary metabolites with a broad range of biological activities. Their antioxidant, anti-inflammatory, and anti-cancer activities make flavonoids widely useful in a variety of industries, including the pharmaceutical and health food industries. However, many flavonoids occur at only low concentrations in plants, and they are difficult to synthesize chemically due to their structural complexity. To address these difficulties, new technologies have been employed to enhance the production of flavonoids *in vivo*. In this study, we used synthetic biology techniques to produce the methylated flavone chrysoeriol in *Nicotiana benthamiana* leaves. The chrysoeriol biosynthetic pathway consists of eight catalytic steps. However, using an *Agrobacterium*-mediated transient expression assay to examine the *in planta* activities of genes of interest, we shortened this pathway to four steps catalyzed by five enzymes. Co-expression of these five enzymes in *N. benthamiana* leaves resulted in *de novo* chrysoeriol production. Chrysoeriol production was unaffected by the *Agrobacterium* cell density used for agroinfiltration and increased over time, peaking at 10 days after infiltration. Chrysoeriol accumulation in agroinfiltrated *N. benthamiana* leaves was associated with increased antioxidant activity, a typical property of flavones. Taken together, our results demonstrate that synthetic biology represents a practical method for engineering plants to produce substantial amounts of flavonoids and flavonoid derivatives without the need for exogenous substrates.

## 1 Introduction

Flavonoids are abundant secondary metabolites produced by plants. To date, more than 10,000 species of plant flavonoids have been identified (Shen et al., 2022; Jiang et al., 2023). These metabolites consist of a 15-carbon skeleton with two aromatic rings (A and B) linked together by a 3-carbonated heterocyclic ring (C), forming a C6-C3-C6 structure. Based on the degree of saturation of ring C, the substitution positions on ring B, and molecular weight, flavonoids can be divided into several subclasses, including flavones, flavonols, anthocyanins, isoflavones, flavanones, and flavanols (Pandey et al., 2016; Xu et al., 2023). Flavonoids protect plants from various abiotic and biotic stresses, such as ultraviolet (UV) radiation and pathogens, respectively, and act as signaling molecules or attract pollinators (Falcone Ferreyra et al., 2012; Shen et al., 2022). Most flavonoids also exhibit diverse biological activities, including antioxidant, anti-inflammatory, antibacterial, and anticancer properties (Kumar and Pandey, 2013; Panche et al., 2016; Liga et al., 2023). Thus, depending on their properties, flavonoids are widely used as raw materials in the pharmaceutical, nutritional, and cosmetic industries (Dias et al., 2021; Jiang et al., 2023). Due to the potential pharmaceutical applications of flavonoids and increasing consumer interest in health foods, the global flavonoid market size is expected to reach USD 3.4 billion by 2031, with a Compound Annual Growth Rate of 6.1% (2023–2031) (Flavonoid Market Report, www.straitsresearch.com). A recent survey indicated that 19 flavonoid-based medicines are on the market following their approval, including 10 natural products from plants and 9 semi-synthetic compounds, indicating that flavonoid production is still heavily dependent on plant extraction (Xu et al., 2023). Although flavonoids are widely distributed in plant tissues, their concentrations tend to be low, limiting the large-scale production of flavonoids *in planta* for industrial use. An alternative approach would be chemical synthesis, but due to the complexity of some flavonoid structures, this would be expensive and time-consuming (Sheng et al., 2020; Sajid et al., 2021; Isogai et al., 2022; Tariq et al., 2023). Therefore, different production systems and approaches are warranted to overcome the hurdles to large-scale flavonoid production.

With the emergence of synthetic biology in the early 2000s, research into the production of plant natural products, including flavonoids and other secondary metabolites, began to attract attention as one of the main targets of this new approach (Zhu et al., 2021). Synthetic biology is the study of the design or synthesis of new biological systems or the redesign of existing systems through a combination of various scientific disciplines, including biology and engineering (Liu and Stewart, 2015). A core concept of synthetic biology is to optimize production of the target compound through repetitive design, build, test, and learn (DBTL) cycles. The results obtained from DBTL cycles are analyzed, standardized, and redesigned for efficiency improvement (Liu et al., 2023). Synthetic biology can be applied to many industries, including medicine, agriculture, food, manufacturing, and fuel production (Voigt, 2020). Microorganisms such as *Escherichia coli* and yeast, as well as plants, can be used as platforms for producing plant natural products. For instance, yeast and plant systems have been successfully utilized to produce artemisinic acid, a precursor of artemisinin used in the production of anti-malarial drugs (Ro et al., 2006; Paddon et al., 2013; Fuentes et al., 2016). In these pilot studies, 25 g L^-1^ and 120 mg kg^-1^ of artemisinic acid were produced in yeast and tobacco (*Nicotiana tabacum*), respectively. Diverse flavonoids, including pinocembrin, naringenin, apigenin, and kaempferol, have been produced using microbial platforms (Isogai et al., 2022; Sun et al., 2022). By contrast, flavonoid production via plant synthetic biology has been relatively under-reported; nevertheless, it has become a rapidly growing field with great potential for enhancing flavonoid production (Jiang et al., 2023; Liu et al., 2023).

Currently, *N. benthamiana* is the most widely used platform in plant synthetic biology. This plant has a relatively short life cycle, produces abundant biomass (100 t ha^-1^), is an economically important non-food plant, and is amenable to transient expression of many different genes of interest (GOI) by *Agrobacterium tumefaciens*-mediated infiltration (agroinfiltration) (Eidenberger et al., 2023; Liu et al., 2023). Transient gene expression by agroinfiltration, in which foreign DNA is temporarily introduced into plant cells without being integrated into the plant genome, is a simple, rapid, highly efficient, and cost-effective method used in plant research (Hwang et al., 2017). Using agroinfiltration, a reconstituted metabolic pathway can easily be transferred into *N. benthamiana* leaves, similar to what can be achieved with microorganism platforms (Lau and Sattely, 2015; Caputi et al., 2018). Moreover, gram-scale production of plant-derived compounds using vacuum-based agroinfiltration can be achieved on a commercial basis (Reed et al., 2017; Reed and Osbourn, 2018). Since 2011, when artemisinin was first produced in *N. tabacum* (Farhi et al., 2011), plant synthetic biology and agroinfiltration have actively been applied to produce various plant natural products, including terpenes (e.g., taxadiene, geraniol, and patchoulol), alkaloids (e.g., catharanthine and tabersonine), and phenylpropanoids (e.g., anthocyanin and antirrhinin) (Lin et al., 2023; Liu et al., 2023).

Chrysoeriol, a 3′-*O*-methoxy flavone derived from luteolin, is produced naturally in several plants including food crops, such as alfalfa (*Medicago sativa*), olive (*Olea europaea*), celery (*Apium graveolens*), perilla (*Perilla frutescens*), pepper (*Capsicum annuum*), mandarin orange (*Citrus reticulata*), and rice (*Oryza sativa*) (Aboulaghras et al., 2022; Flavonoid database at http://koreanfood.rda.go.kr). Chrysoeriol exhibits various pharmacological effects that are beneficial for human health, such as antioxidant, anti-inflammatory, anticancer, antidiabetic, antibacterial, antifungal, and neuroprotective properties (Aboulaghras et al., 2022). In plants, chrysoeriol biosynthesis begins with phenylalanine, which is produced by the shikimate pathway. Phenylalanine is converted to *p*-coumaroyl-CoA through the phenylpropanoid pathway, which involves sequential catalysis by three enzymes: phenylalanine ammonia-lyase (PAL), cinnamate-4-hydroxylase (C4H), and 4-coumaryl-CoA ligase (4CL). Next, *p*-coumaroyl-CoA is combined with three molecules of malonyl-CoA by chalcone synthase (CHS) to produce naringenin chalcone. Naringenin chalcone is subsequently converted to naringenin, which serves as an important intermediate of several flavonoid subclasses, by chalcone isomerase (CHI). For flavone biosynthesis, naringenin is further desaturated to apigenin by flavone synthase (FNS), and apigenin is hydroxylated to luteolin by a flavonoid 3′ hydroxylase (F3′H). Subsequently, chrysoeriol is formed from luteolin by *O*-methyltransferase (OMT) (Lam et al., 2015; Gong et al., 2024). To date, the key enzymes involved in each step of the chrysoeriol biosynthetic pathway—PAL, C4H, 4CL, CHS, CHI, FNS, F3′H, and OMT—have been identified in various plants (Huang et al., 2010; Ogo et al., 2013; Lam et al., 2014; Falcone Ferreyra et al., 2015; Lam et al., 2015; Park et al., 2016; Rea et al., 2019; Liu et al., 2020). However, chrysoeriol production in plants using metabolic engineering or synthetic biology has not been reported.

Here, we used synthetic biology to produce chrysoeriol in a plant platform. We simplified the natural biosynthetic pathway of chrysoeriol to four steps, phenylalanine to naringenin to apigenin to luteolin to chrysoeriol, using five enzymes. We then selected the genes encoding these enzymes with optimal activity in *N. benthamiana* based on data obtained through DBTL cycles. Subsequently, we created a multigene vector by assembling five transcription units in a single T-DNA region of a binary vector. We introduced this vector into tobacco (*N. benthamiana* or *N. tabacum*) using transient and stable transformation and examined chrysoeriol production. We successfully produced chrysoeriol in tobacco through plant synthetic biology, highlighting the potential to produce valuable flavonoids and their derivatives in plant platforms.

## 2 Materials and methods

### 2.1 Plant materials and growth conditions

Two tobacco platforms (*N. benthamiana* and *N. tabacum* L. cv. Xanthi) were used in this study. Seeds were sown in horticultural soil, and the plants were grown in a greenhouse at 25°C under long-day conditions (16 h/8 h, light/dark). The third to fourth leaves from the tops of tobacco plants grown for approximately 3 to 4 weeks were used for *Agrobacterium*-mediated transient expression assays.

### 2.2 Generation of DNA modules

Eight GOI involved in the chrysoeriol metabolic pathway were selected from various plants, including *Arabidopsis thaliana* (At), *O. sativa* (Os or R), maize (*Zea maize*; Zm), *Cannabis sativa* (Cs), and *C. reticulata* (Cr), based on previous reports (**Table 1**). The native coding sequences (CDSs) of seven GOI, excluding *AtPAL*, were converted to tobacco codon-optimized sequences without type IIS restriction enzyme (*Bsa*I and *Bpi*I) sites using the GenScript GenSmart™ Codon Optimization tool for efficient expression in tobacco plants (**Supplementary Table 1**). Codon optimization adjusted the GC content to 41.2-49.1%, making it more suitable for dicots. Additionally, the optimized sequences showed a similarity of 70-77% to the original sequences.

**Table 1:** List of genes used in this study.

Step	Pathway	Gene	No	Name	Accession Number	CDS size (bp)	References
1	Phenylalanine → Naringenin	PAL	1	AtPAL	NM_129260	2,178	Huang et al., 2010
		CHS	2	OsCHS	NM_001423410	1,197	Ogo et al., 2013
2	Naringenin → Apigenin	FNS	3-1	OsFNS	AK100972	1,551	Lam et al., 2014
			3-2	ZmFNSI	NM_001157695	1,011	Falcone Ferreyra et al., 2015; Zhu et al., 2020
3	Apigenin → Luteolin	F3’H	4	OsF3’H	AK064736	1,581	Shih et al., 2008; Lam et al., 2015; Park et al., 2016
4	Luteolin → Chrysoeriol	OMT	5-1	CsOMT21	JP459899	1,113	Rea et al., 2019
			5-2	ROMT9	AK061859	870	Kim et al., 2006; Lam et al., 2015
			5-3	CrOMT2	XM_006441201	1,101	Liu et al., 2020

At, *Arabidopsis thaliana*; Os or R, *Oryza sativa*; Zm, *Zea maize*; Cs, *Cannabis sativa*; Cr, *Citrus reticulate*.

For module assembly, universal plant DNA parts were obtained from Addgene (MoClo Tool Kit #1000000044, MoClo Plant Parts Kit #1000000047, MoClo Plant Parts II and Infrastructure Kit #1000000135, Cambridge, MA, USA) (Weber et al., 2011; Engler et al., 2014; Gantner et al., 2018). To create a level 0 module, synthetic CDSs of eight selected genes were amplified with the primer sets listed in **Supplementary Table 2**, and each CDS PCR product was assembled into level 0 acceptors (**Supplementary Table 3**) as described in Marillonnet and Grützner (2020). Transcriptional units (level 1) were assembled using the level 0 DNA parts (promoters, CDSs, terminators, acceptors) listed in **Supplementary Table 3**. Finally, for multigene expression, level M modules were constructed by combining level 1 modules, an end-linker, and a level M vector. The accuracy of the newly generated modules was checked by restriction enzyme digestion and sequence analysis.

### 2.3 *Agrobacterium*-mediated transient expression in *N. benthamiana*

Level 1 and level M modules were transformed into *Agrobacterium tumefaciens* strain GV3101 using the freeze-thaw method (Weigel and Glazebrook, 2006). Transformed *Agrobacterium* cells were inoculated into liquid YEB medium containing antibiotics and grown overnight at 28°C. The cells were then diluted (1/1,000) in 10 mL of liquid YEB medium and incubated to a cell concentration of OD_600_ 1.0. Cultured cells were harvested and re-suspended in infiltration medium (5% D-glucose, 10 mM MES, 10 mM MgCl_2_, 200 μM acetosyringone) to OD_600_ 0.8. After 2 h of incubation in the dark, the *Agrobacterium* suspension was infiltrated into the abaxial surfaces of *N. benthamiana* leaves using a needleless 1 mL syringe, and the agroinfiltrated leaves were sampled after 6 days. At this time, *Agrobacterium* cells containing the *p19* gene, which helps suppress RNA silencing and stabilizes the expression of foreign genes (Jay et al., 2023), were co-transfected into *N. benthamiana* leaves at the same concentration. For the substrate feeding assays, substrates (naringenin, apigenin, luteolin) diluted in infiltration medium at a concentration of 100 μM were injected into the agroinfiltrated leaves five days after the initial agroinfiltration using a syringe, and the leaves were collected 1 day later. Naringenin, apigenin, and luteolin were purchased from Sigma Aldrich (MO, USA). The leaves were either ground in liquid nitrogen for RNA extraction or freeze-dried for flavonoid analysis. Infiltration medium lacking *Agrobacterium* or *Agrobacterium* cells harboring the empty vector were infiltrated into *N. benthamiana* leaves as a negative control.

### 2.4 Stable transformation of *N. tabacum*

Stable transformation of *N. tabacum* was conducted using the leaf disk method (Gallois and Marinho, 1995). Briefly, *N. tabacum* seeds were surface sterilized by washing with 70% ethanol for 1 min, 25% bleach for 15 min, and distilled water 3–5 times. The sterilized seeds were sown in germination medium, and plants were grown for 4 to 6 weeks under long-day conditions (28°C, 16 h/8 h, light/dark). For one iteration of the experiment, 100 N*. tabacum* leaf discs were collected, submerged in *Agrobacterium* cells harboring the BAR+PCFF’O level M vector for 20 min, and co-cultivated for 2 days in tobacco callus induction (TCI) medium (28°C, dark). After co-cultivation, the leaf discs were transferred onto TCI selection medium containing phosphinothricin (PPT, 10 mg L^-1^) and incubated at 28°C (dark) for 7 days to select for callus resistant to PPT. About 50 calli were induced and they were transferred to tobacco shoot induction medium, and shoot formation was induced in the light. The 20 regenerated shoots were transferred to hormone-free MS medium (root induction medium) to induce root formation, and the 11 plants that formed roots were acclimated to soil. The acclimated *N. tabacum* plants were transferred to the greenhouse and cultivated to maturity. A total of 23 transgenic *N. tabacum* plants (T_0_ generation) were obtained through repeated experiments and were used for further studies. To examine the integration of the construct in the *N. tabacum* genome, genomic DNA was extracted from the leaves of 6-week-old plants using cetyltrimethylammonium bromide extraction solution (Biosolution, Suwon, Korea). Genomic PCR was conducted in a BIO-RAD C1000™ Thermal cycler (Bio-Rad, Hercules, CA, USA) using BAR-specific primer sets (listed in **Supplementary Table 2**).

### 2.5 RNA isolation and quantification of transcript levels

Total RNA was isolated from pulverized tobacco leaves (100 mg) using a QIAGEN RNeasy Plant Mini Kit (Qiagen, Hilden, Germany), according to the manufacturer’s instructions. cDNA was synthesized from 2 µg of total RNA using TOPscript™ RT DryMIX (dT18) (Enzynomics, Daejeon, Korea), and reverse-transcription quantitative PCR (RT-qPCR) was performed to measure gene expression levels using a CFX96™ Real-Time System (Bio-Rad) with TOPreal™ SYBR Green qPCR High-ROX PreMIX (Enzynomics). Protein phosphatase 2A (*PP2A*) was used as an internal control (Liu et al., 2012). Primer sequences are shown in **Supplementary Table 2**.

### 2.6 Flavonoid isolation and high-performance liquid chromatography

Flavonoids were extracted from agroinfiltrated leaves or the leaves of transgenic plants (10-20 mg) using 80% (v/v) methanol with shaking at 4°C overnight. To analyze flavonoid glycosides, following centrifugation (13,000 g, 10 min, 4°C), the supernatant was concentrated under a nitrogen stream and re-suspended in 100 μL 80% (v/v) methanol. For flavonoid aglycones, the methanol (80%, v/v) extract was incubated with 1N HCl for 2 h at 94°C, and hydrolyzed flavonoids were fractionated with ethyl acetate. The supernatant was transferred into a new tube and evaporated under a nitrogen stream. The concentrated extracts were dissolved in 100 μL 80% (v/v) methanol.

Quantification of flavonoids was performed using an HPLC (LC-20A HPLC system, Shimadzu, Kyoto, Japan) instrument equipped with a Symmetry C18 column (100Å, 5 µm, 4.6 mm X 250 mm, Waters Corp., Milford, MA, USA). The mobile phase consisted of water containing 0.1% formic acid (A) and acetonitrile containing 0.1% formic acid (B) and was used at a flow rate of 1 mL min^–1^. The binary gradient method was used as follows: 5–55% B (0–30 min), 55–100% B (30–35 min), 100–5% B (35–37 min), and 5% B (37–42 min). A 10 μL sample of extract was injected into the system, and the column temperature was set at 30°C. A UV/diode-array detector (DAD) was used to record the spectra of flavonoids between 190 and 800 nm. Chromatograms of naringenin and flavones (apigenin, luteolin, chrysoeriol) were analyzed at 288 nm and 350 nm, respectively. The chrysoeriol aglycone content was calculated using the equation [x=(y+1252)/5E+06, R^2^ = 1] obtained by calibration curves of a chrysoeriol standard (PhytoLab GmbH & Co. KG, Vestenbergsgreuth, Germany). In the equation, x is content (μg/g) of chrysoeriol and y is peak area detected by HPLC analysis.

### 2.7 Qualitative analysis of chrysoeriol aglycone and chrysoeriol glucoside

Qualitative analysis of flavonoids was conducted using a quadrupole time-of-flight mass spectrometer (QToF-MS) (SCIEX Co., Framingham, MA, USA) equipped with an ultra-performance liquid chromatography-diode array detector (UPLC-DAD) system (SCIEX Co.). A 1 μL of sample was injected and separated in a reversed-phase-column (CORTECS UPLC T3, 2.1×150 mm I.D., 1.6 μm, Waters Co., Milford, MA, USA) coupled with a CORTECS UPLC VanGuard™ T3 pre-column (2.1×50 mm I.D., 1.6 μm, Waters Co.). The column temperature was set at 30°C, and the flow rate was 0.3 mL min^–1^. The mobile phase, consisting of water containing 0.5% (v/v) formic acid (Sol. A) and acetonitrile containing 0.5% (v/v) formic acid (Sol. B), was used with the following program: 25% B (20 min), 25–50% B (20–25 min), 50–90% B (25–30 min), 90% B (30–32 min), 90–5% B (32–35 min), and 5% B (35–40 min). The mass spectra of chrysoeriol aglycone and chrysoeriol glycoside were measured between *m/z* 100–1,200 in positive ionization mode. Mass spectrometric conditions were maintained as described in Lee et al. (2024). Chrysoeriol-7-O-glucoside (ChemFaces, Wuhan, China) was used as a standard.

### 2.8 Antioxidant activity assay

Total antioxidant activity was examined by colorimetric methods using an OxiTec™ DPPH (2,2-Diphenyl-1-picrylhydrazyl) Antioxidant Assay Kit (BO-DPH-200, BIOMAX, Seoul, Korea) and an OxiTec™ Total Antioxidant Capacity (TAC) Assay Kit (BO-TAC-200, BIOMAX) according to the manufacturer’s protocols. The flavonoid aglycones extracted by the above-mentioned method were diluted 20-fold with 80% (v/v) methanol and then used as the sample solution, and the absorbance was measured at 517 nm for the DPPH assay and 450 nm for the TAC assay. Antioxidant activity of *N. benthamiana* leaf extracts containing chrysoeriol was further validated by ABTS (2,2’-azinobis(3-ethylbenzothiazoline-6-sulfonic acid)) radical cation decolorization assay (Re et al., 1999). Briefly, ABTS radical cation (ABTS^•+^) solution produced by oxidation of ABTS with potassium persulfate was stored in the dark at refrigerator for at least 12 h. The absorbance of ABTS^•+^ solution was adjusted with water to 0.7 at 734 nm. The 10 μL diluted leaf extract was mixed with 190 μL ABTS^•+^ solution and allowed to react for 30 min in the dark, then the absorbance was measured at 734nm. 10 μL 80% methanol was used as a solvent blank. ABTS radical scavenging activity (%) was calculated using the formula, % = [1-(A-A’)/(B-B’)]X100, where, A is the absorbance derived from mixing ABTS^•+^ solution with leaf extract; A’ is the absorbance obtained by mixing water with leaf extract; B is the absorbance of the mixture of ABTS^•+^ solution and 80% methanol; B’ is the absorbance of the mixture of water and 80% methanol. Trolox was used as standard substance.

### 2.9 Statistical analysis

Data were analyzed using IBM SPSS Statistic ver. 26 (IBM SPSS Inc., Armonk, NY, USA). One-way ANOVA followed by a Tukey’s HSD (honestly significant difference) test was performed to determine whether there was a significant difference between samples. The significance level was set to 0.05 (*P*-value < 0.05). All data are expressed as means with standard error (SE).

## 3 Results

### 3.1 Reconstruction of the chrysoeriol biosynthetic pathway

**Figure 1:**
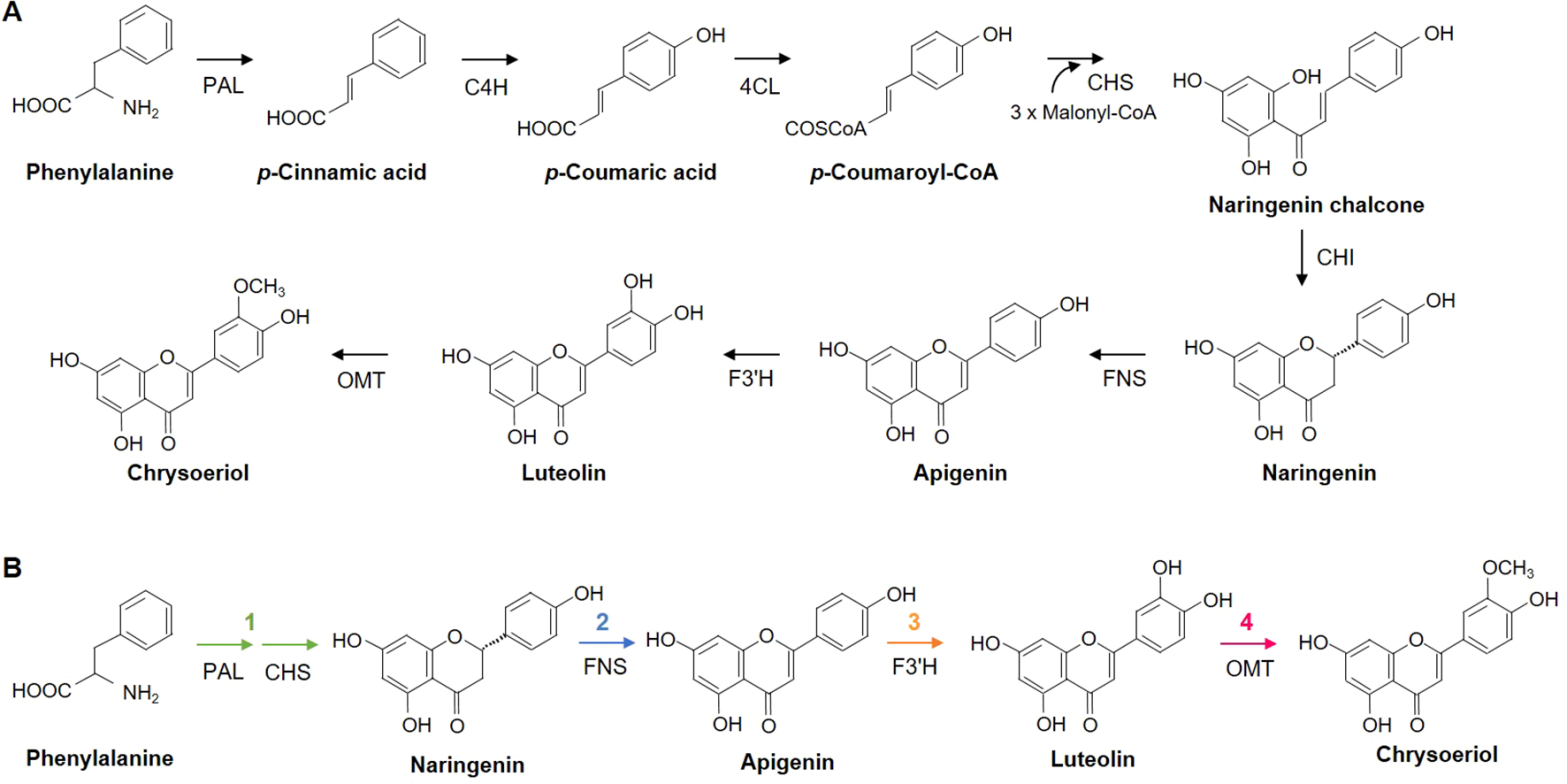
Reconstruction of a chrysoeriol biosynthetic pathway. **(A)** Typical eight-step biosynthetic pathway of chrysoeriol from phenylalanine. **(B)** Re-designed four-step chrysoeriol biosynthetic pathway requiring only five enzymes: PAL, CHS, FNS, F3′H, and OMT. PAL, phenylalanine ammonia lyase; C4H, cinnamic acid 4-hydroxylase; 4CL, 4-coumaric acid: CoA ligase; CHS, chalcone synthase; CHI, chalcone isomerase; FNS, flavone synthase; F3′H, flavonoid 3′ hydroxylase; OMT, O-methyltransferase.

The chrysoeriol biosynthetic pathway is known to require eight enzymatic reactions (**Figure 1A**). However, Ogo et al. (2013) showed that rice co-expressing the *PAL* and *CHS* genes could accumulate naringenin, suggesting that the number of enzymes required to perform the step in the naringenin biosynthetic pathway from phenylalanine could be reduced from five to two. Therefore, we redesigned the chrysoeriol biosynthetic pathway, reducing it to four steps catalyzed by five enzymes: PAL, CHS, FNS, F3’H, and OMT (**Figure 1B**).

Based on previous reports, we selected eight GOI from various plant species covering the functions required to redesign the chrysoeriol biosynthetic pathway (**Table 1**; **Figure 1B**). For the first step, we selected *AtPAL* and *OsCHS* from *A. thaliana* and *O. sativa*, respectively, for naringenin biosynthesis. For the second step, we chose *OsFNS* from *O. sativa* and *ZmFNSI* from *Z. mays* for apigenin production. For the third step, we selected *OsF3’H* from *O. sativa* for luteolin biosynthesis. For the final step, we chose three additional genes for chrysoeriol biosynthesis: *CsOMT21* (*C. sativa*), *ROMT9* (*O. sativa*), and *CrOMT2* (*C. reticulata*).

**Figure 2:**
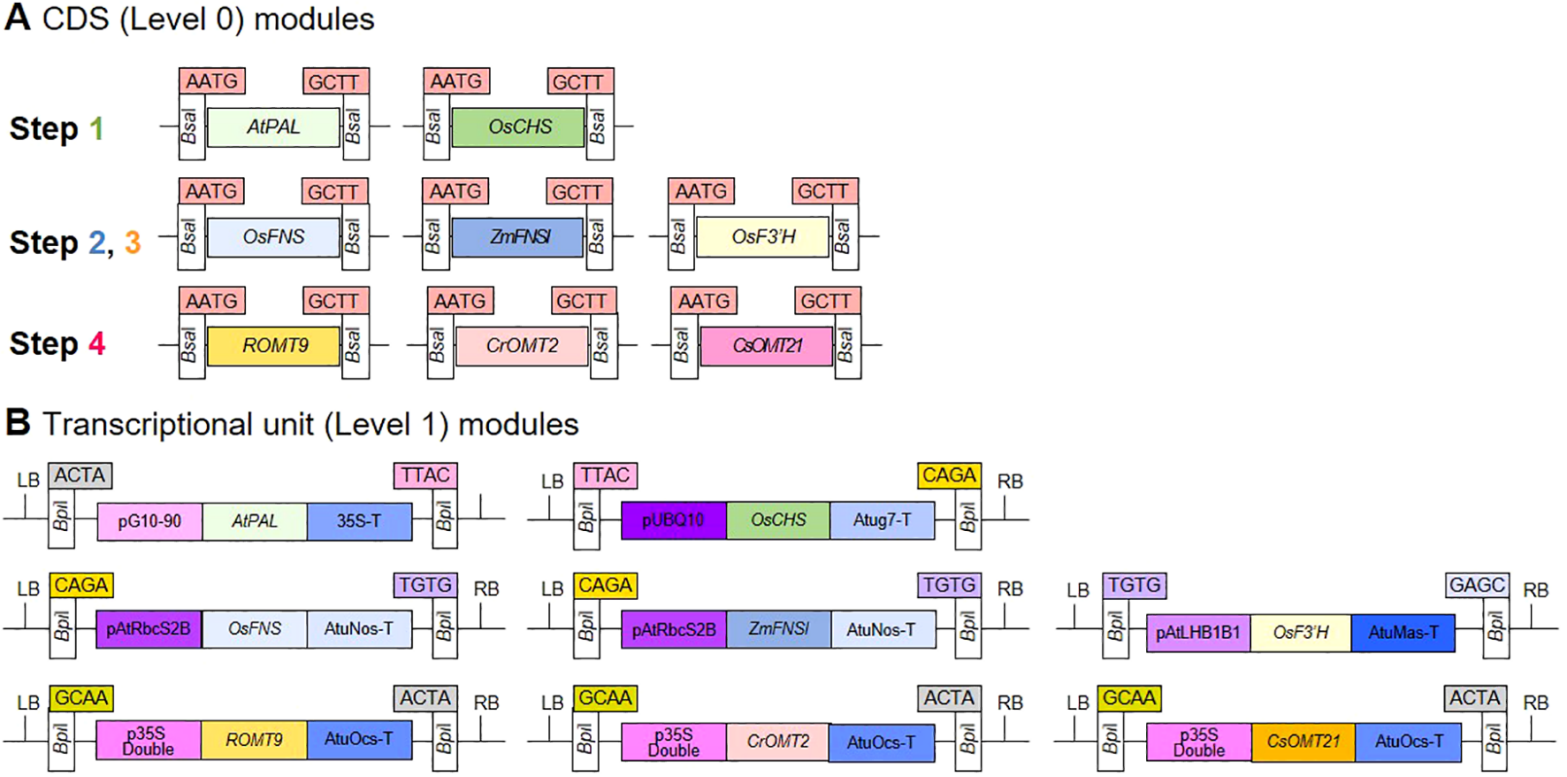
Generation of DNA modules for transient expression in *N. benthamiana.* **(A, B)** Schematic diagrams of synthetic gene modules of interest created using the modular cloning (MoClo) system. Level 0 modules contain the coding sequences (CDSs) of the target genes, while Level 1 modules consist of a promoter, CDS, and terminator for constitutive overexpression. Atug7-T, 3’UTR and polyadenylation signal/terminator of *Atug7* (*A. tumefaciens*); AtuNos-T, 3’UTR and polyadenylation signal/terminator of *nos* (*A. tumefaciens*); AtuMas-T, 3’UTR and polyadenylation signal/terminator of *mas* (*A. tumefaciens*); AtuOcs-T, 3’UTR and polyadenylation signal/terminator of *ocs* (*A. tumefaciens*); 35S-T, 3’UTR and polyadenylation signal/terminator of *35s* (Cauliflower Mosaic Virus); pG10-90, promoter G10-90; pUBQ10, ~800 bp fragment upstream of Arabidopsis *UBQ10*; pAtRbcS2B, promoter and 5’ UTR of *RbcS2B* (AT5g38420, *A. thaliana*); pAtLHB1B1, promoter and 5’ UTR of *LHB1B1* (AT2g34430, *A. thaliana*); p35S Double, promoter (double) of *35s* and 5’UTR (Tobacco Mosaic Virus); LB, left border; RB, right border. Each module has specific 4-bp overhangs produced by *Bsa*I or *Bpi*I restriction enzymes for module assembly.

We generated level 0 modules harboring the synthetic CDSs of the eight GOI to test for enzymatic activity (**Figure 2A**). We then assembled the eight transcriptional units using the level 0 DNA parts, including a promoter, CDS, terminator, and acceptor, to create transcription units that could be heterologously expressed in plants (**Figure 2B**; **Supplementary Table 3**).

### 3.2 Production of flavonoid aglycones in *N. benthamiana* by transient expression

We investigated the activities of the enzymes encoded by the GOI by infecting *N. benthamiana* leaves with *Agrobacterium* cells that harbored each level 1 module along with the appropriate substrate. At 6 days after infiltration (DAI), we isolated total RNA from the *N. benthamiana* leaves and examined the transcript levels of the eight GOI by RT-qPCR (**Supplementary Figure 1**). The expression levels of all tested genes increased compared to control leaves infiltrated with infiltration medium (NC), suggesting that the genes were transiently overexpressed through agroinfiltration.

**Figure 3:**
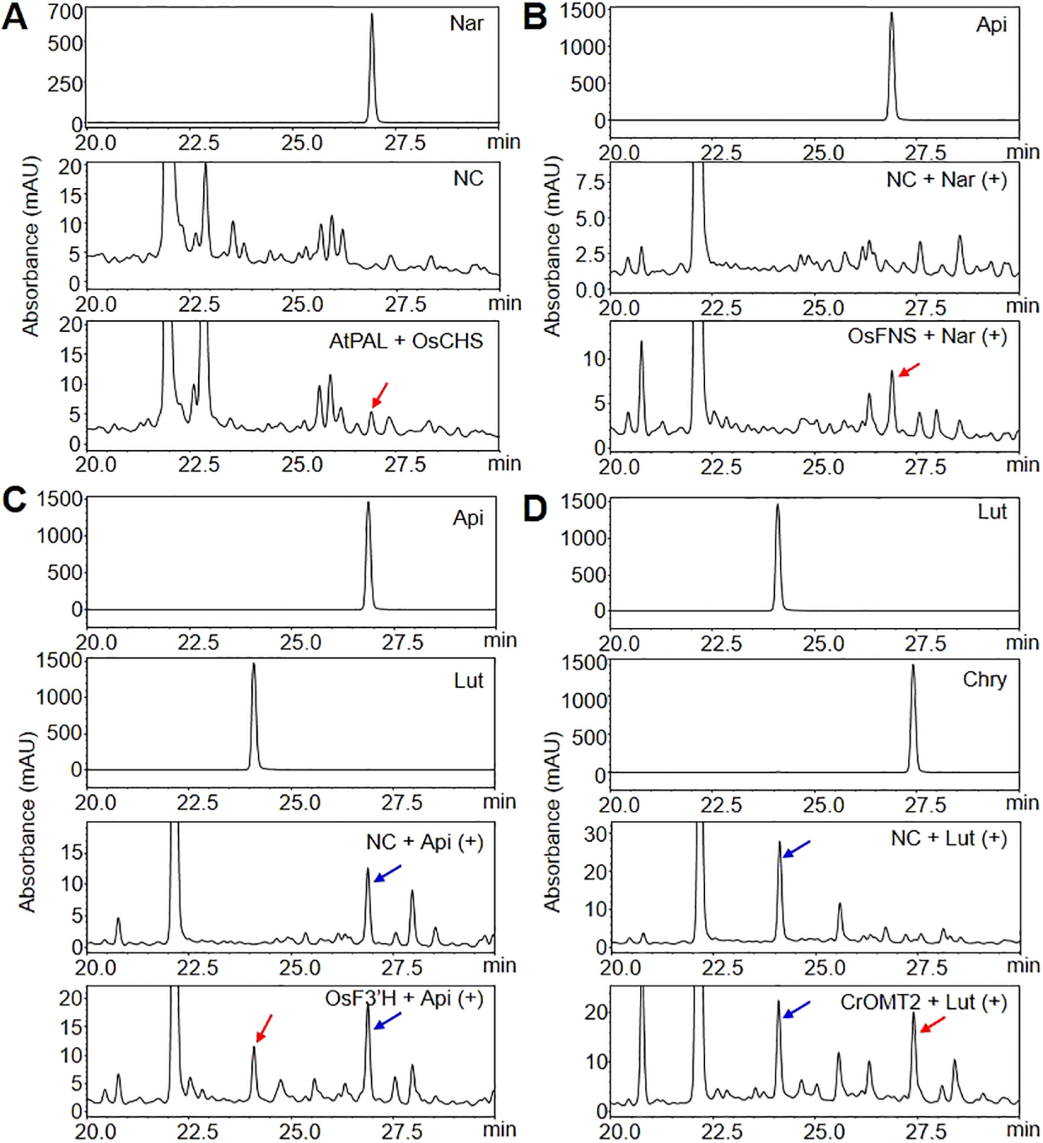
Production of flavonoids in *N. benthamiana* leaves transiently expressing genes of interest (GOI). *Agrobacterium* cells harboring a single level 1 module were infiltrated into *N. benthamiana* leaves. Naringenin (Nar), apigenin (Api), or luteolin (Lut) were infiltrated into the same abaxial leaves as a substrate 5 days after the initial infiltration, followed by incubation for 24 h. Infiltration medium was injected into *N. benthamiana* leaves as a negative control (NC). Flavonoids were extracted from lyophilized *N. benthamiana* leaves with 80% methanol and analyzed by HPLC. Peaks were detected at 288 nm for naringenin **(A)** and at 350 nm for flavones (apigenin, luteolin, and chrysoeriol) **(B-D)**. The peaks were identified by comparing relative retention times and UV spectra to the standards. Peaks corresponding to flavonoids produced by the transient expression of *AtPAL* and *OsCHS*, *OsFNS, OsF3’H*, or *CrOMT2* are indicated by red arrows. Substrates were marked with blue arrows. In **(B)**, a naringenin substrate detecting at 288nm was displayed in **Supplementary Figure 2**.

The investigate whether the expression of GOI can produce flavonoids at each step of chrysoeriol biosynthesis in plants, we performed HPLC to measure the flavonoid aglycones extracted from agroinfiltrated *N. benthamiana* leaves. When *AtPAL* and *OsCHS* were co-expressed, naringenin was produced, which is consistent with previous reports (**Figure 3A**). However, no naringenin was detected in control leaves injected with infiltration medium, suggesting that the newly produced naringenin accumulated due to the simultaneous expression of *AtPAL* and *OsCHS* in *N. benthamiana* leaves. For the second step, the expression of *OsFNS* resulted in apigenin formation when leaves were supplied with naringenin (**Figure 3B**; **Supplementary Figure 2**), whereas *N. benthamiana* leaves expressing *ZmFNSI* showed no evidence of FNS enzyme activity (**Supplementary Figure 2**). Luteolin production was achieved by expressing *OsF3’H* in leaves supplied with the substrate apigenin (**Figure 3C**; **Supplementary Figure 3**). For the final step, the conversion of luteolin into chrysoeriol, we tested the enzymatic activities of the proteins encoded by three different genes. Chrysoeriol was produced only when *CrOMT2* was expressed along with the substrate luteolin (**Figure 3D**; **Supplementary Figure 4**), whereas *N. benthamiana* leaves heterologously expressing *ROMT9* or *CsOMT21* were unable to convert luteolin to chrysoeriol (**Supplementary Figure 4**). In addition, we detected no chrysoeriol in leaves infiltrated with infiltration medium or in agroinfiltrated leaves that were not provided with substrate (**Figure 3**; **Supplementary Figures 2**-**4**). Based on these results, we selected five essential enzymes with confirmed functions for the redesign of the chrysoeriol biosynthetic pathway: AtPAL, OsCHS, OsFNS, OsF3’H, and CrOMT2.

### 3.3 Chrysoeriol production by multi-gene co-expression in *N. benthamiana* leaves

**Figure 4:**
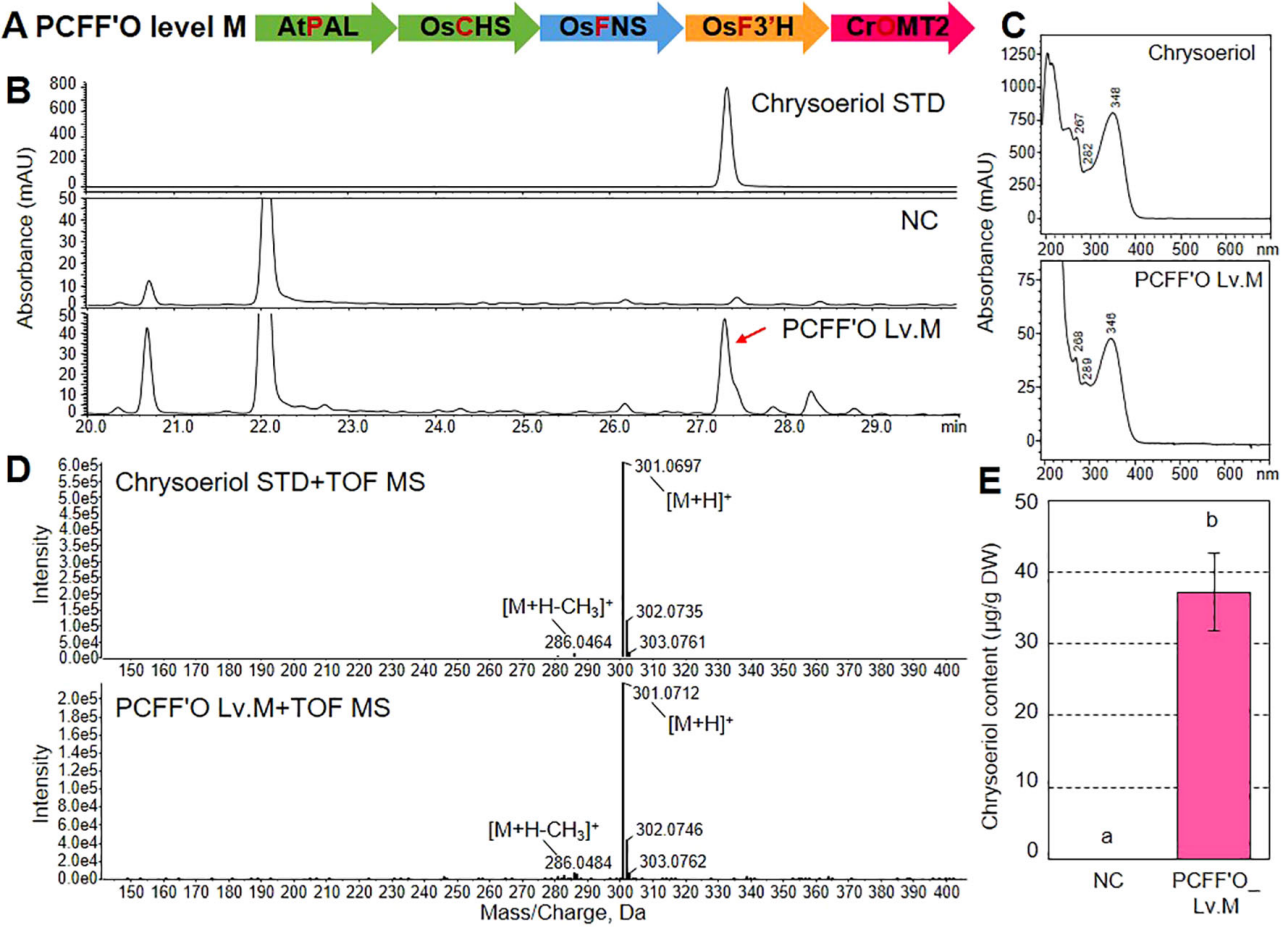
Production of chrysoeriol aglycone and glycoside by a multi-gene expression vector in *N. benthamiana* leaves. **(A)** Schematic diagram of the multigene expression vector (PCFF’O level M). This vector was generated by assembling five transcription units into a single binary vector. *Agrobacterium* cells harboring the multigene vector were infiltrated into *N. benthamiana* leaves. **(B)** HPLC chromatograms of chrysoeriol aglycone produced in *N. benthamiana* leaves expressing five genes: *AtPAL*, *OsCHS*, *OsFNS*, *OsF3’H*, and *CrOMT2* (PCFF’O). Chrysoeriol aglycone was detected at 350 nm. The peak corresponding to chrysoeriol aglycone produced by transiently expressing the five genes is indicated by a red arrow. NC, negative control (*N. benthamiana* leaves infiltrated with infiltration medium). **(C, D)** UV spectrum **(C)** and QToF-MS fragmentation **(D)** of the chrysoeriol standard peak and the peak detected in *N. benthamiana* leaves transfected with the PCFF’O level M vector. **(E)** Total content of chrysoeriol aglycone produced in *N. benthamiana* leaves transfected with infiltration medium or *Agrobacterium* cells harboring the PCFF’O level M vector. Bars indicate standard error (SE) of six technical replicates from two independent experiments. Different letters represent significant differences based on ANOVA with Tukey HSD (*P* < 0.05).

We investigated whether the heterologous co-expression of *AtPAL, OsCHS*, *OsFNS*, *OsF3’H*, and *CrOMT2* in *N. benthamiana* leaves would lead to chrysoeriol production. First, we assembled the transcription units of the five genes in a single binary vector, resulting in a multigene vector capable of simultaneously expressing *At* ***P*** *AL*, *Os* ***C*** *HS*, *Os* ***F*** *NS*, *Os* ***F*** *3* ***’*** *H*, and *Cr* ***O*** *MT2* (named the PCFF’O level M vector; **Figure 4A**). To test whether the PCFF’O level M vector could confer chrysoeriol production in a plant platform, we infiltrated *Agrobacterium* cells carrying the PCFF’O level M vector into *N. benthamiana* leaves. RT-qPCR analysis showed that the five genes were stably expressed in *N. benthamiana* leaves transformed with the PCFF’O level M vector, but not in leaves infiltrated with infiltration medium (**Supplementary Figure 5**). When flavonoids were subsequently analyzed by HPLC, extracts from leaves agroinfiltrated with the PCFF’O level M vector had a distinct peak at UV 350 nm with the same retention time (RT=27.3 min) as the chrysoeriol standard, which was not detected in *N. benthamiana* leaves infiltrated with infiltration medium (**Figure 4B**). In addition, the observed peaks were identified as chrysoeriol aglycones based on their UV spectra and QToF-MS patterns. Specifically, their UV absorption maxima (λmax) were 268, 289, and 346 nm (**Figure 4C**), and the peak showed MS fragmentation patterns with a [M+H–CH_3_]^+^ ion at *m/z* 286.0484 and a [M+H]^+^ ion at *m/z* 301.0712, which are identical to those of the chrysoeriol aglycone standard (**Figure 4D**). Finally, the total chrysoeriol aglycone content produced in *N. benthamiana* via agroinfiltration of the redesigned chrysoeriol biosynthetic pathway was 37.2 μg g^–1^ dry weight (DW) based on quantitative analysis (**Figure 4E**).

Since flavonoids in plants are mainly present in the form of glycosides (Dias et al., 2021), we examined the types of chrysoeriol glycosides produced in *N. benthamiana* leaves using UPLC-DAD-QToF-MS. The extracted-ion chromatogram (XIC) at *m/z* 301.07, which is molecular ion of protonated chrysoeriol aglycone, shows two peaks in *N. benthamiana* leaves transfected with the PCFF’O level M vector (**Supplementary Figure 6A**). MS/MS spectrum for major peak detected at 28.168min was consistent with the fragmentation patterns of chrysoeriol-7-*O*-glucoside standard ([M+H-Glu]^+^ at *m/z*: 301.0709, and [M+H]^+^ at *m/z*: 463.1233). The peak detected at 29.098min was identified as chrysoeriol-7-O-(6”-malonyl-glucoside), a structure in which a malonyl group is attached to chrysoeriol-7-*O*-glucoside ([M+H-Mal-Glu]^+^ at *m/z*: 301.0707, [M+H-Mal]^+^ at *m/z*: 463.1190, and [M+H]^+^ at *m/z*: 549.1223) (**Supplementary Figure 6B**).

### 3.4 Optimal conditions for chrysoeriol production in tobacco

**Figure 5:**
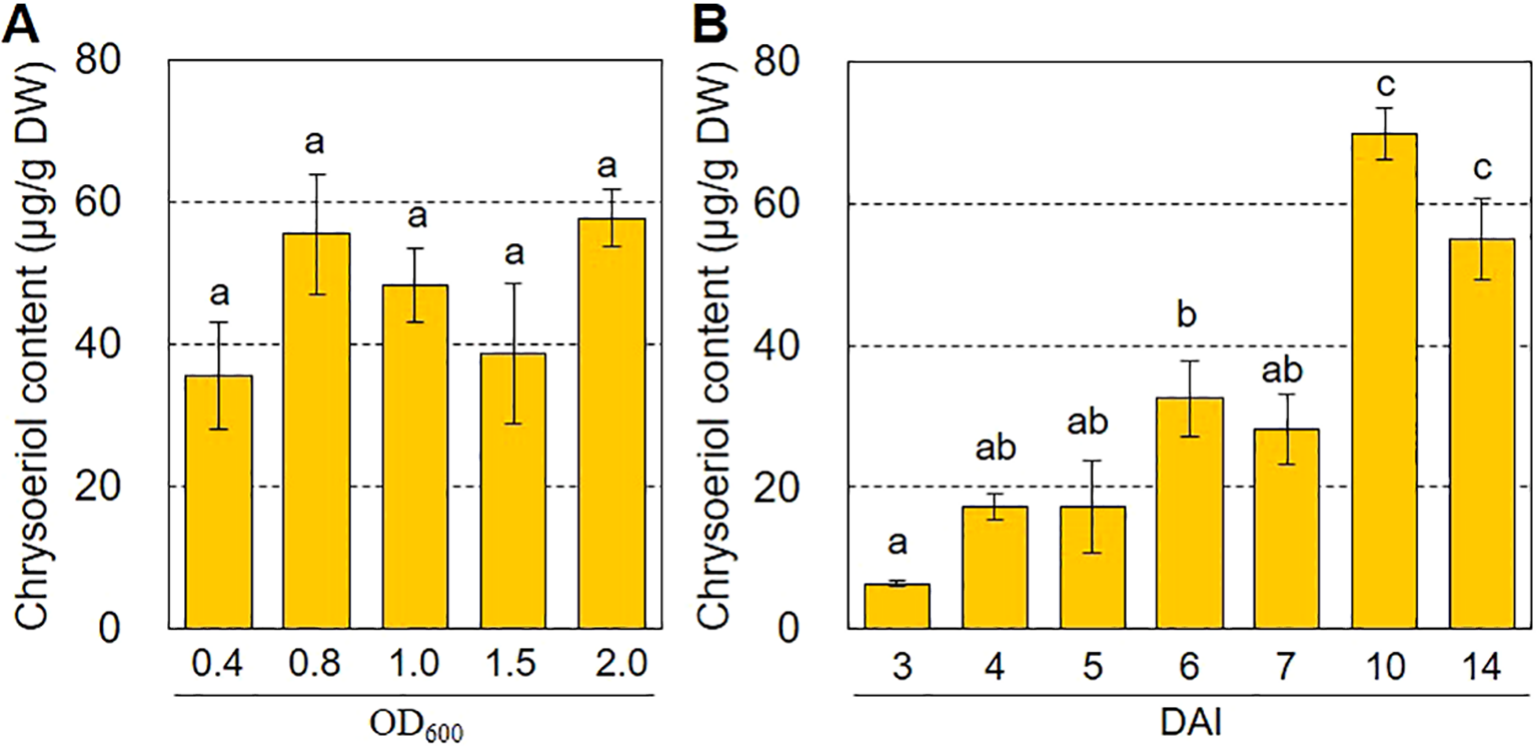
Optimal conditions for increasing chrysoeriol production in *N. benthamiana* leaves. **(A)** Effects of *Agrobacterium* concentration on chrysoeriol production. *Agrobacterium* cells harboring the PCFF’O level M vector were grown to OD_600_ ranging from 0.4 to 2.0 and infiltrated into *N. benthamiana* leaves. Leaves were harvested at 6 DAI, and the chrysoeriol content was measured from six technical replicates of two independent experiments. Bars indicate standard error (SE). **(B)** Effects of harvest time on chrysoeriol production. *Agrobacterium* cells harboring the PCFF’O level M vector at a concentration of OD_600_ of 0.8 were infiltrated into *N. benthamiana* leaves. Three different leaves were sampled at 3, 4, 5, 6, 7, 10, and 14 DAI. Statistical analysis was performed using the results of three replicates by one-way ANOVA and Tukey HSD test. Differences were considered significant when *P* < 0.05. Bars indicate standard error (SE).

The efficiency of agroinfiltration depends on many factors. The agroinfiltration conditions must be optimized to maximize production of the target compound (Kaur et al., 2021). To explore the optimal conditions for chrysoeriol production via agroinfiltration, we investigated how chrysoeriol accumulation was affected by the *Agrobacterium* cell density used for agroinfiltration and by the incubation period after agroinfiltration. As shown in **Figure 5A**, *Agrobacterium* cell density (OD=0.4–2.0) had no significant effect on the production of chrysoeriol aglycone. However, the content of chrysoeriol aglycone increased over time after infiltration (DAI 3 to 10), reaching approximately 69.78 μg g^-1^ DW at 10 DAI (**Figure 5B**). This level is 10.8-times higher than that at 3 DAI and 2.1-times higher than that at 6 DAI. Overall, these results indicate that the incubation period after agroinfiltration is an important factor to optimize for maximal production of the target compound.

**Figure 6:**
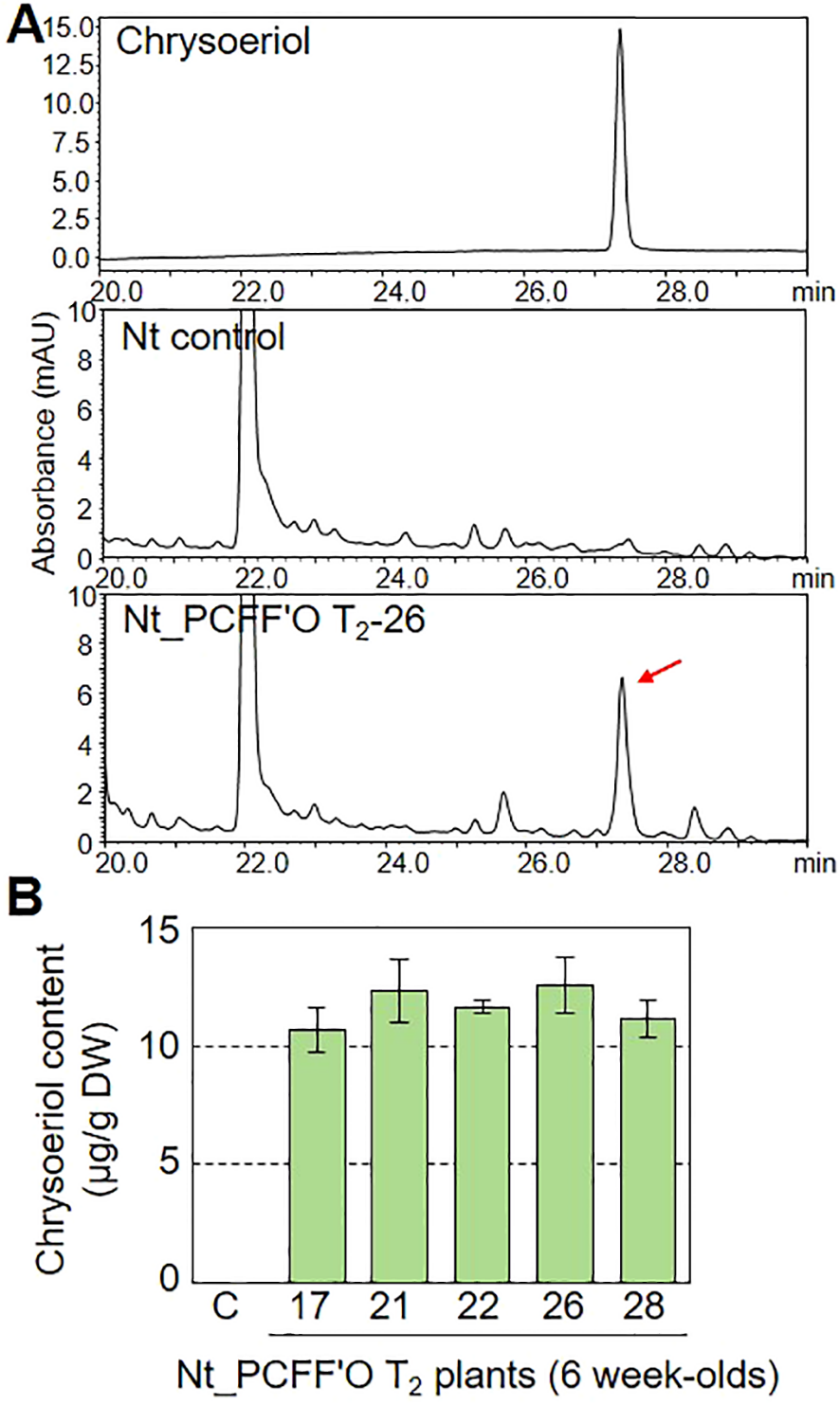
Verification of chrysoeriol production by stably transformed plants. **(A)** HPLC chromatograms of chrysoeriol aglycone produced in *N. tabacum* transgenic plants expressing five genes: *AtPAL*, *OsCHS*, *OsFNS*, *OsF3’H*, and *CrOMT2* (Nt_PCFF’O, T_2_ generation). Chrysoeriol produced in the leaves of a transgenic plant is indicated by a red arrow. **(B)** Total content of chrysoeriol aglycone produced in leaves of *N. tabacum* transgenic plants expressing the five genes. Bars indicate standard error (SE) of technical replicates (n=4). C, non-transgenic plant. Different letters represent significant differences based on ANOVA with Tukey HSD (*P* < 0.05).

To compare the efficiency of chrysoeriol production in transiently versus stably transformed plants, we generated transgenic *N. tabacum* (Nt) plants. *N. tabacum* is widely used for stable transformation due to its extremely high biomass (Fresquet-Corrales et al., 2017; Ikram and Simonsen, 2017; Stephenson et al., 2020). We generated the BAR+PCFF’O level M multigene vector, which includes an basta-resistance gene (*BAR*) along with the PCFF’O genes, and confirmed its ability to confer chrysoeriol production in agroinfiltrated *N. benthamiana* leaves (**Supplementary Figure 7**). We then obtained transgenic *N. tabacum* plants by infecting leaf discs with *Agrobacterium* cells containing the BAR+PCFF’O level M vector. Analysis of flavonoids in extracts from mature leaves of 6-week-old T_1_ generation transgenic plants revealed that chrysoeriol aglycone accumulated only in the *N. tabacum* transformants (Nt PCFF’O; **Supplementary Figure 8A**). We performed qualitative and quantitative analysis of flavonoids extracted from mature leaves of 20 T_1_ generation *N. tabacum* transgenic plants and selected 14 T_1_ transformants containing chrysoeriol aglycone (**Supplementary Figure 8C**). We then collected T_2_ generation seeds from the Nt PCFF’O T_1_-2 plant with the highest chrysoeriol aglycone content. Chrysoeriol aglycone was detected in 35 T_2_ generation *N. tabacum* transgenic plants (**Supplementary Figure 8D**). The transgenic *N. tabacum* plants containing chrysoeriol grew and developed normally and were indistinguishable from non-transgenic plants (**Supplementary Figures 8B, E**). The introduction of the *BAR* gene was confirmed in the five T_2_ generation *N. tabacum* transgenic plants with the highest chrysoeriol aglycone contents, and all five PCFF’O genes were highly expressed in T_2_ generation *N. tabacum* transgenic plants (**Supplementary Figure 9**). Furthermore, approximately 12.59 μg g^-1^ DW of chrysoeriol aglycone was produced in T_2_ generation *N. tabacum* transgenic plants (**Figure 6**). Based on these results, although both transient and stable plant transformation can result in chrysoeriol production, the amount of chrysoeriol produced in a short period (~10 days) via transient expression was approximately 5.5-times higher than that produced in stably transformed plants.

### 3.5 Antioxidant activity of chrysoeriol aglycone produced in *N. benthamiana*

**Figure 7:**
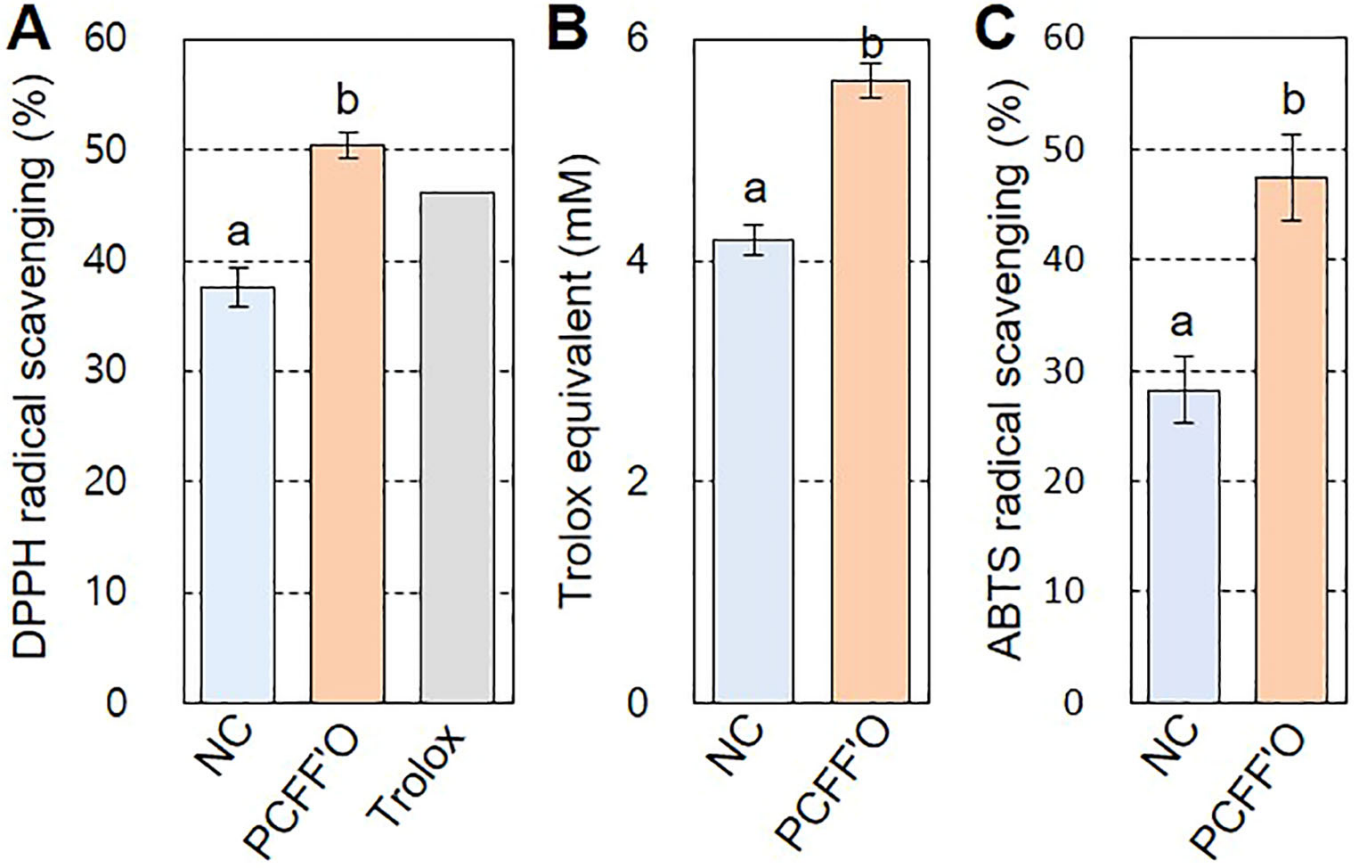
Antioxidant activity of *N. benthamiana* leaf extracts containing chrysoeriol. Flavonoid was extracted with 80% Me-OH from *N. benthamiana* leaves infiltrated with infiltration medium (NC) or *Agrobacterium* cells harboring the PCFF’O level M vector (PCFF’O). Flavonoids extracted from three independent leaves were used for the antioxidant activity assays. **(A)** DPPH radical scavenging activity. Trolox (80 μg mL^-1^), a water-soluble vitamin E analog, was used as the positive control. **(B)** TAC assay. Total antioxidant activity is represented as Trolox equivalent antioxidant capacity. Bars indicate standard error (SE) of two independent experiments (NC; *n*=10, PCFF’O; *n*=30). **(C)** ABTS radical scavenging activity. Result is means ± SE from triplicate experiments (n=3). Different letters represent significant differences based on ANOVA with Tukey HSD (*P* < 0.05).

Since antioxidant activity is a well-known biological property of flavones (Shen et al., 2022), we conducted DPPH, TAC, and ABTS assays to investigate the antioxidant activity of chrysoeriol aglycone produced in *N. benthamiana* leaves. DPPH radicals showed 37.58% inhibition by treatment with flavonoid extract from *N. benthamiana* leaves infiltrated with infiltration medium, whereas a higher inhibition rate (50.45%) was observed for *N. benthamiana* leaf extract infiltrated with the PCFF’O level M vector (**Figure 7A**). This activity is higher than the 46.06% DPPH radical inhibition shown by Trolox, a water-soluble vitamin E analog used as a positive control. In the TAC assay, which measures the efficiency of copper(II) oxidation to copper(I) by antioxidants, higher antioxidant activity was observed in PCFF’O leaf extracts, which accumulated more chrysoeriol aglycone than extracts from control leaves (**Figure 7B**). The antioxidant activity measured by ABTS was observed to be 1.7 times higher in *N. benthamiana* leaf extract infiltrated with the PCFF’O level M vector than in the control (**Figure 7C**). These results demonstrate that synthetic biology techniques enhance the accumulation of the flavone chrysoeriol in leaves, thereby boosting its antioxidant activity.

## 4 Discussion

Recent advances in biotechnology and bioinformatics have facilitated DNA synthesis and have led to the development of numerous databases containing genomics and metabolomics data. As the functions of genes involved in the biosynthesis of plant natural products have become well understood, it has become possible to mine metabolic pathways to produce valuable products by combining key genes from various plants (Maeda, 2019). These advances have driven the development of synthetic biology, which enables the production of high-value materials from plants (Liu et al., 2023). In this study, we demonstrated that chrysoeriol production could be achieved by redesigning the chrysoeriol metabolic pathway and introducing it into tobacco using a plant synthetic biology approach. Specifically, we (1) simplified the chrysoeriol biosynthetic pathway; (2) selected five essential enzymes and generated a multi-gene expression vector via DBTL cycles; and (3) successfully produced chrysoeriol in *N. benthamiana* or *N. tabacum* using transient or stable transformation methods, respectively. 

Synthetic biology approaches and microbial platforms have been widely used for the production of three intermediates in the chrysoeriol biosynthetic pathway: one flavanone (naringenin) and two flavones (apigenin and luteolin) (Pandey et al., 2016; Sheng et al., 2020; Isogai et al., 2022). For example, *E. coli* was engineered to produce 1,073 mg L^-1^ naringenin from glucose and tyrosine (Wu et al., 2021), and an engineered yeast strain produced 1,129 mg L^-1^ naringenin from glucose (Zhang et al., 2021). The reconstruction of an apigenin biosynthetic pathway in *E. coli* led to the production of 30 mg L^-1^ apigenin from *p*-coumaric acid (Lee et al., 2015). An engineered strain of the bacterium *S. albus* achieved the *de novo* biosynthesis of 0.09 mg L^-1^ luteolin using glucose as a carbon source (Marín et al., 2017). In general, naringenin production in microbial platforms is accomplished through the expression of genes encoding four enzymes: TAL, 4CL, CHS, and CHI. The naringenin can then be converted to apigenin and luteolin by the additional expression of *FNS* and *F3′H*, respectively. In plants, the accumulation of naringenin (~70 μg g^-1^ DW) or apigenin (~120 μg g^-1^ DW) was observed in rice seeds co-expressing *PAL* and *CHS* or *PAL, CHS*, and *FNS*, respectively (Ogo et al., 2013). In the present study, we successfully produced naringenin by co-expressing *PAL* and *CHS* and apigenin or luteolin by single-gene expression of *FNS* or *F3′H*, respectively, in tobacco when the appropriate substrate was supplied (**Figure 3**). We hypothesized that utilizing genes encoding key enzymes to engineer a shortened biosynthetic pathway would decrease the size of the vector required to transform plant cells, thereby enhancing the efficiency of multi-gene expression in plants.

Wu et al. (2023) produced 85 mg L^-1^ chrysoeriol from luteolin in *E. coli* using ROMT9 engineering. However, there are no previous reports of the *de novo* production of chrysoeriol in microorganisms or plants via a reconstructed metabolic pathway. Here, we report the *de novo* production of chrysoeriol via a reconstituted chrysoeriol biosynthetic pathway in plants without exogenous substrates (**Figure 4**). The use of expensive substrates poses a significant challenge to the industrial production of flavonoids (Koopman et al., 2012). Therefore, we believe that the capacity of plant synthetic biology to produce chrysoeriol without requiring additional substrates is a major advantage. Although microorganisms can easily produce a wide range of high-value compounds in large quantities, plants have the advantage that the expression of membrane protein such as CYP450 is more efficient (Maeda, 2019). In addition, plant-derived natural products are less toxic in plants when produced by metabolic engineering. For these reasons, we believe that our redesigned chrysoeriol pathway may have enabled the *de novo* production of chrysoeriol in plants, and this is the first successful example of its production in plants.

Chrysoeriol biosynthesis follows the general flavonoid biosynthetic pathway in plants, and is produced by methylation of luteolin: (1) naringenin → apigenin → luteolin → chrysoeriol (**Figure 1**). However, in some plants, it has been suggested that luteolin can be converted from eriodictyol, which is formed from naringenin by flavonoid 3′-hydroxylase (F3′H), via flavone synthase (FNS) (MetaCyc Metabolic Pathway Database, www.metacyc.org). This route is proposed to be: (2) naringenin → eriodictyol → luteolin → chrysoeriol (Muruganathan et al., 2022; Deng et al., 2024). Here, we chose the conversion of apigenin to luteolin through the action of F3’H as the pathway for chrysoeriol biosynthesis because flavones tend to be more abundant in plant species than flavanones and they are end-products of flavonoids. Since the chrysoeriol pathway is not active in tobacco leaves, engineering the pathway using the optimal genes for chrysoeriol and intermediate biosynthesis identified in other plants is one of the easily accessible strategies for synthetic biology. Chrysoeriol has been produced in various plants. However, genetic information on chrysoeriol-producing plants is still lacking. Therefore, flavonoid biosynthesis genes have been selected from well-characterized plants, and these genes have been widely used for reconstruction of metabolic pathway (Isogai et al., 2022). In this study, we selected *CHS, FNS, F3′H*, and *OMT* genes with reported enzymatic activities from *O. sativa, C. sativa*, and *C. reticulata* that produce chrysoeriol, and designed metabolic pathways using *PAL* and *FNS* genes from *A. thaliana* and *Z. mays*, respectively (**Table 1**). Among the F3′H candidate genes, both OsF3′H enzymes, OsCYP75B3 or OsCYP75B4, convert apigenin to luteolin (Park et al., 2016). OsCYP75B3 showed higher preference for apigenin than naringenin, dihydrokaempferol, and kaempferol. OsCYP75B4 has a substrate preference for apigenin and kaempferol, but it additionally shows chrysoeriol 5′-hydroxylation activity converting chrysoeriol to selgin (Lam et al., 2015). Therefore, we chose the *OsCYP75B3* as F3′H candidate gene. Although OsCYP75B3 has a substrate preference for naringenin, eriodictyol was not detected in *N. benthamiana* leaves infiltrated with *Agrobacterium* cells harboring the PCFF’O level M vector. In addition, *A. thaliana* transgenic plants overexpressing *OsFNS* produce apigenin, luteolin, and chrysoeriol (Lam et al., 2014). These data support that the chrysoeriol pathway proposed in **Figure 1** is a feasible and suitable pathway in *N. bentamiana* and *N. tabacum*.

In a previous study, ZmFNS1 was shown to synthesize apigenin from naringenin in *E. coli* and *A. thaliana* (Falcone Ferreyra et al., 2015). ROMT9 and CsOMT21 also converted luteolin to chrysoeriol in *E. coli* (Kim et al., 2006; Rea et al., 2019). Nevertheless, we did not observe any activity of these enzymes in *N. benthamiana*, even though we optimized the codons and provided substrates (**Supplementary Figures 2**, **4**). In synthetic biology, the same enzymes can show different activities in different heterologous hosts, depending on host-specific enzyme efficiency and the mechanisms regulating foreign gene expression (Cravens et al., 2019; Chan et al., 2023). Therefore, the selection of host-optimized enzymes or the optimization of their activity is an important factor in enhancing the production of target compounds.

Engineering a metabolic pathway requires the expression of multiple enzymes, posing a major challenge to the successful production of target compounds. In plants, multiple gene expression can easily be accomplished by agroinfiltration using different strategies. These strategies include co-transformation, which utilizes a mixture of *Agrobacterium* strains, each harboring a single-gene expression vector, and transformation with a single *Agrobacterium* strain carrying a multigene vector containing multiple transcription units linked in a single T-DNA region (Reed and Osbourn, 2018; Zhu et al., 2020). In this study, transformation with a multigene vector resulted in more chrysoeriol production (37.2 μg g^-1^, DW) compared to co-transformation with multiple single-gene vectors (4.3 μg g^-1^, DW) (**Figure 4**; **Supplementary Figure 10**). The co-introduction of multiple genes into the same plant cell and uniform expression of the multiple genes are the key aspects of any agroinfiltration strategy. Although co-transformation can readily be used to test engineered metabolic pathways, the ratio and density of mixed *Agrobacterium* cells can affect the co-expression of multiple genes in a same plant cell (Carlson et al., 2023). Also, the increased *Agrobacterium* cell density required to transfer multiple single-gene vectors can limit the production of target compounds by agroinfiltration (Reed and Osbourn, 2018). Indeed, we found that the expression levels of the five genes were lower following co-transformation of different strains of *Agrobacterium* harboring single-gene vectors compared to using multi-gene expression vectors (**Supplementary Figures 5**, **10D**). These results demonstrate that multi-gene vectors are required for stable, high gene expression levels, an important factor in enhancing chrysoeriol production.

We found that the transient expression method produced 5.5-times more chrysoeriol than that produced by stable transformation (**Figures 4**, **6**). Interestingly, the chrysoeriol content produced by stable transformation varied depending on the position of the leaf (**Supplementary Figure 11**). When we analyzed chrysoeriol content in young and mature leaves of 6-week-old transgenic tobacco plants, chrysoeriol content was 35 μg g^-1^ (DW) in very early young leaves (1^st^ leaf). It was 4.6 times higher than in mature leaves (2^nd^ to 5^th^ leaves). This was similar to the chrysoeriol content produced by transiently expression in *N. benthamiana* leaves (37.2 μg g^-1^, DW). In contrast, mature leaves accumulated 5-10 μg g^-1^ (DW) chrysoeriol. We though that these results may be due to metabolic flux of chrysoeriol or its intermediates to other pathways or degradation of chrysoeriol not originally present in tobacco. Also, this may be due to the use of strong and constitutive promoters, and it can be overcome by using inducible promoters. Future studies will be required to verify this.

The production of plant secondary metabolites is influenced by various factors, including environmental stresses or chemical elicitors (Wang et al., 2015; Humbal and Pathak, 2023). Although it has not yet been reported that chrysoeriol production is enhanced by these factors, we have observed that chrysoeriol production can be increased approximately 2-fold by treatment of tobacco transgenic plants with methyl jasmonate and sucrose (data not shown). This suggests that chrysoeriol production can be increased by other factors in addition to methyl jasmonate and sucrose, and we believe that this strategy can be applied to the scale-up of chrysoeriol production in the future.

Both transient expression and stable transformation methods can be effective in the production of high value compounds and have the potential for industrial scale (Kallam et al., 2023). Transient expression can provide a rapid process with high copy numbers, resulting in the production of high yields per biomass production. It also minimizes the biosafety and regulatory issues associated with open-field cultivation (Owen et al., 2017; Kaur et al., 2021). However, large-scale transient expression typically requires more complicated infrastructure than the cultivation of transgenic plants and may not yield high biomass because transient expression is less efficient in mature plants. Stable transformation takes a long time to select highly efficient transgenic lines, but once they are selected, we can get a lot of biomass at once with minimal labor, and we can provide large amounts of biomass on demand while preserving it as seed. Although the economics of scale-up production can vary depending on the target compounds, we believe that inducing production a short time after introducing the pathway is the optimal method to produce chrysoeriol. Ultimately, initial validation is critical step for plant synthetic biology technologies to choose the suitable plant species and right methods for the commercial scale production of target compounds.

In summary, the use of a redesigned biosynthetic pathway for chrysoeriol in *N. benthamiana* leaves using synthetic biology techniques enabled the production of up to 69.78 μg g-1 DW of chrysoeriol. This level is 23 times higher than the chrysoeriol content of brown rice (3 μg g-1 DW) and similar to that of red pepper (58 μg g-1 DW) (http://koreanfood.rda.go.kr). Our system can also be used to express additional genes involved in flavonoid modifications, such as acylation, glycosylation, and prenylation, to produce chrysoeriol derivatives.

## Data Availability

The datasets presented in this study can be found in online repositories. The names of the repository/repositories and accession number(s) can be found in the article/**Supplementary Material**.
