# Supplementary material for: Engineering Nicotiana benthamiana for chrysoeriol production using synthetic biology approaches
Source: Front Plant Sci. 2024 Dec 17;15:1458916. doi: 10.3389/fpls.2024.1458916 (PMC11685227; doi:10.3389/fpls.2024.1458916)
Supplement: Supplementary file 1 [file SupplementaryFile1.docx]

Supplementary Material

# Supplementary Figures and Tables

## Supplementary Figures


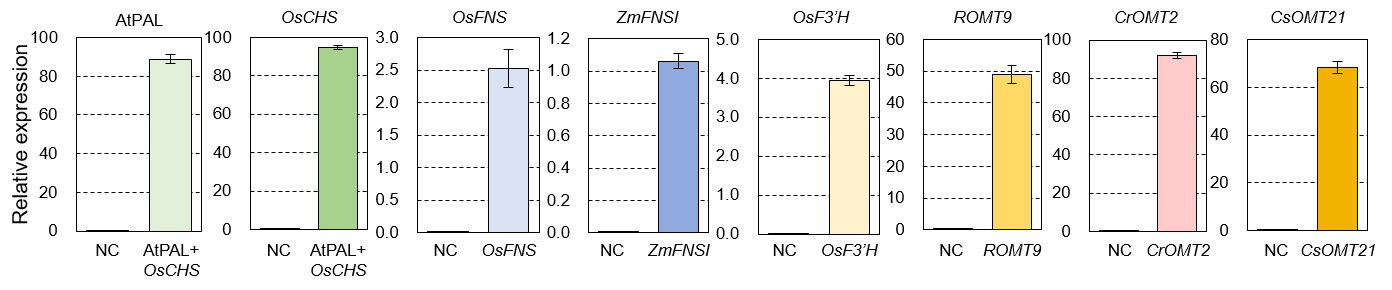


**Supplementary Figure 1.** Expression levels of eight genes of interest (GOI) in *N. benthamiana* leaves. Agrobacterium cells harboring a single level 1 module were infiltrated into abaxial leaves of *N. benthamiana*. Infiltration medium was used as a negative control (NC). Total RNA was isolated at 6 days after infiltration (DAI), and RT-qPCR was conducted using gene-specific primer sets. Protein phosphatase 2A (*PP2A*) was used as an internal control. Bars indicate standard error (SE) of three technical replicates.


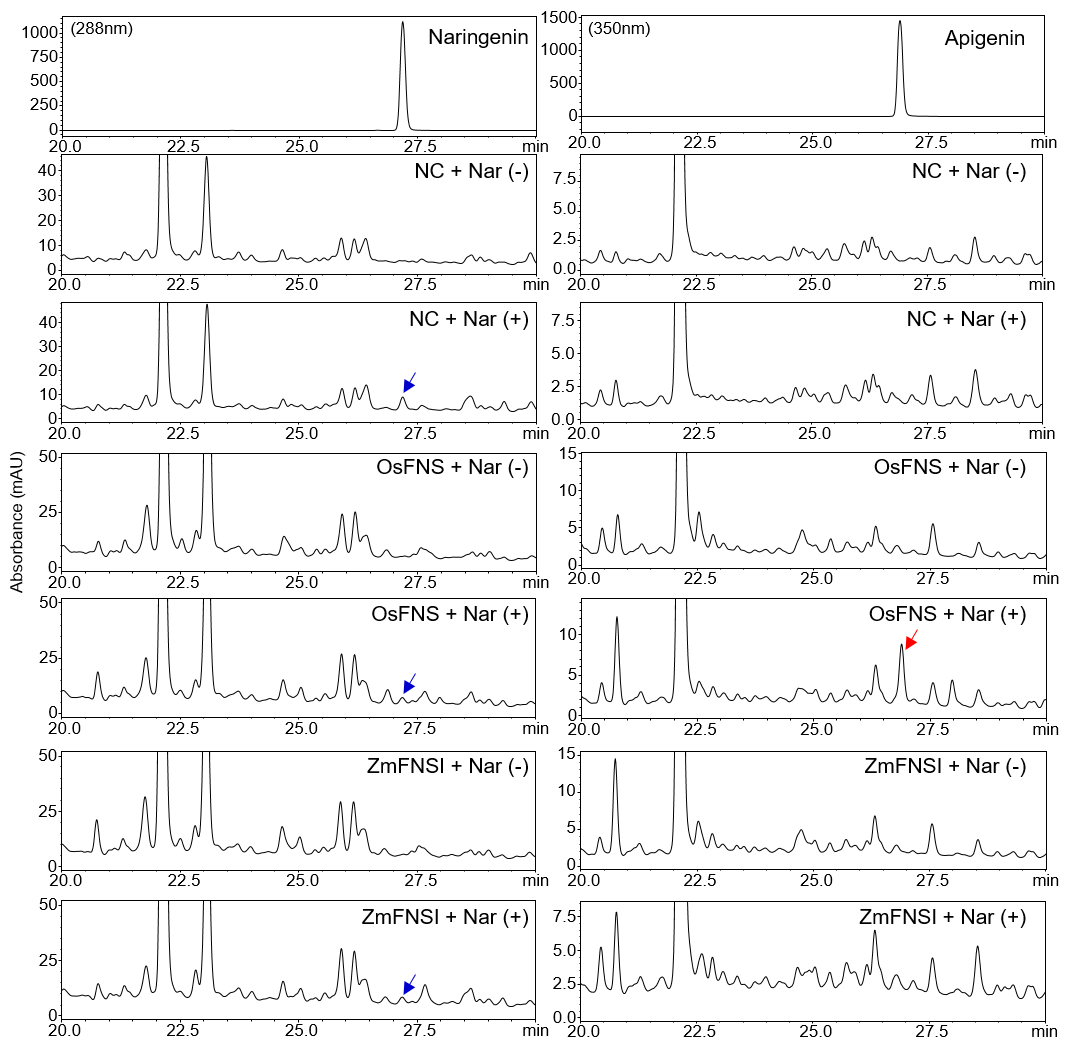


**Supplementary Figure 2.** Analysis of FNS activity for apigenin production in *N. benthamiana.* HPLC chromatograms of apigenin aglycone produced in *N. benthamiana* leaves expressing *OsFNS* or *ZmFNSI*. Naringenin (blue arrow) was added as a substrate. Apigenin peaks are indicated by a red arrow.


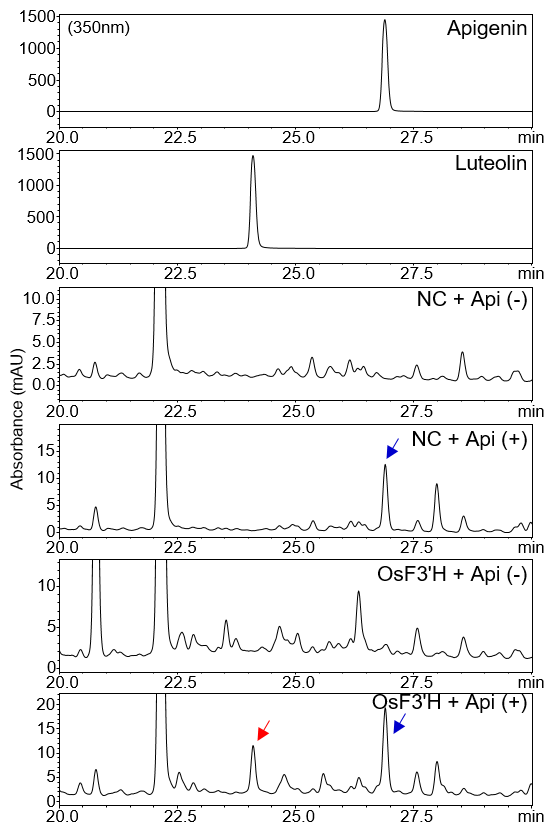


**Supplementary Figure 3.** Analysis of OsF3'H activity for luteolin production in *N. benthamiana.* HPLC chromatograms of luteolin aglycone produced in *N. benthamiana* leaves heterologously expressing *OsF3'H*. Apigenin (blue arrow) was added as a substrate. Luteolin peaks are indicated by a red arrow.


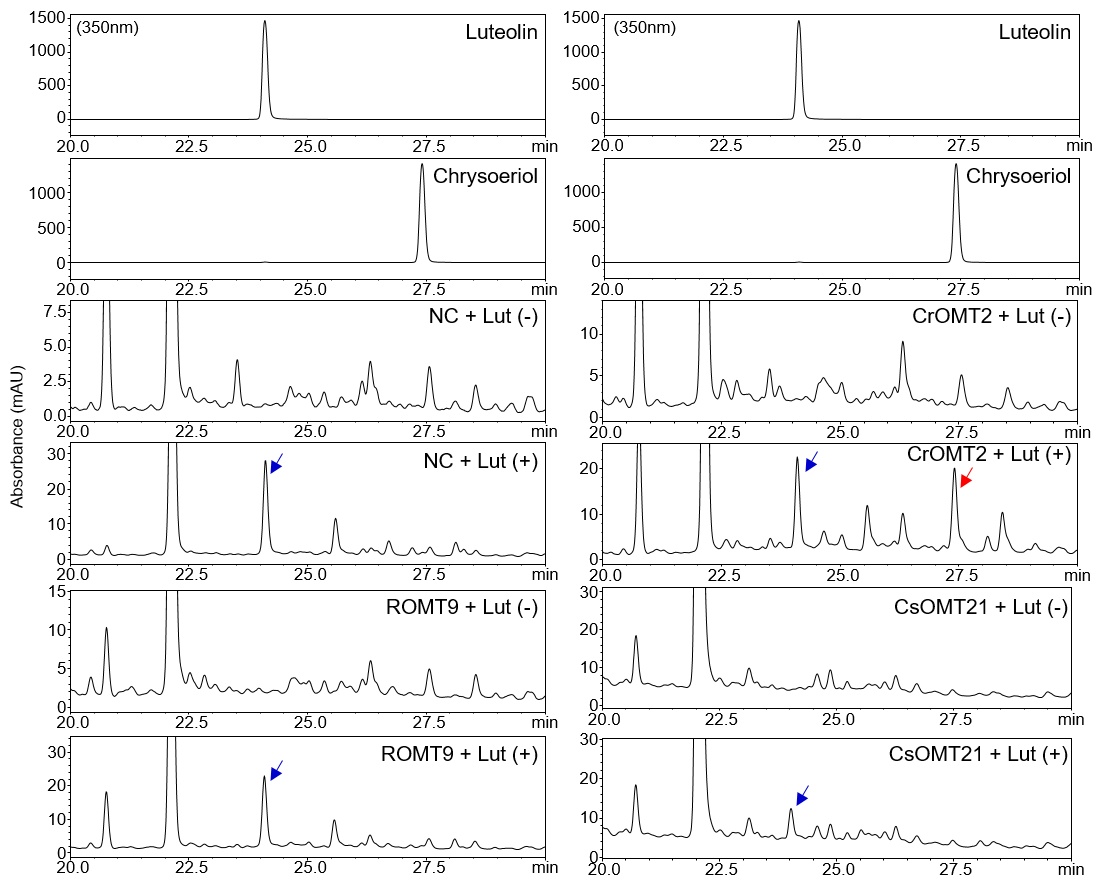


**Supplementary Figure 4.** Analysis of OMT activity for chrysoeriol production in *N. benthamiana.* HPLC chromatograms of chrysoeriol aglycone produced in *N. benthamiana* leaves heterologously expressing *ROMT9, CrOMT2,* or *CsOMT21*. Luteolin (blue arrow) was added as a substrate. Chrysoeriol peaks are indicated by a red arrow.


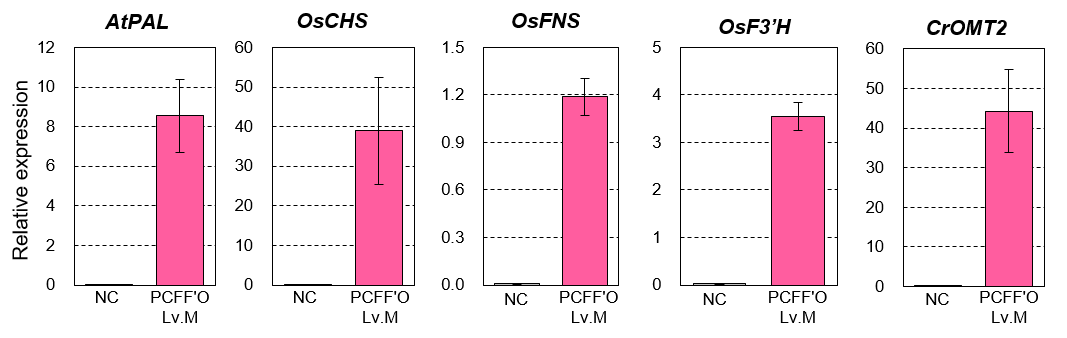


**Supplementary Figure 5.** Expression levels of five GOI in *N. benthamiana* leaves following agroinfiltration of the multigene expression vector. Infiltration medium was used as a negative control (NC). Total RNA was isolated at 6 days after infiltration (DAI), and RT-qPCR was conducted using gene-specific primer sets. *PP2A* was used as an internal control. Bars indicate standard error (SE) of three technical replicates.


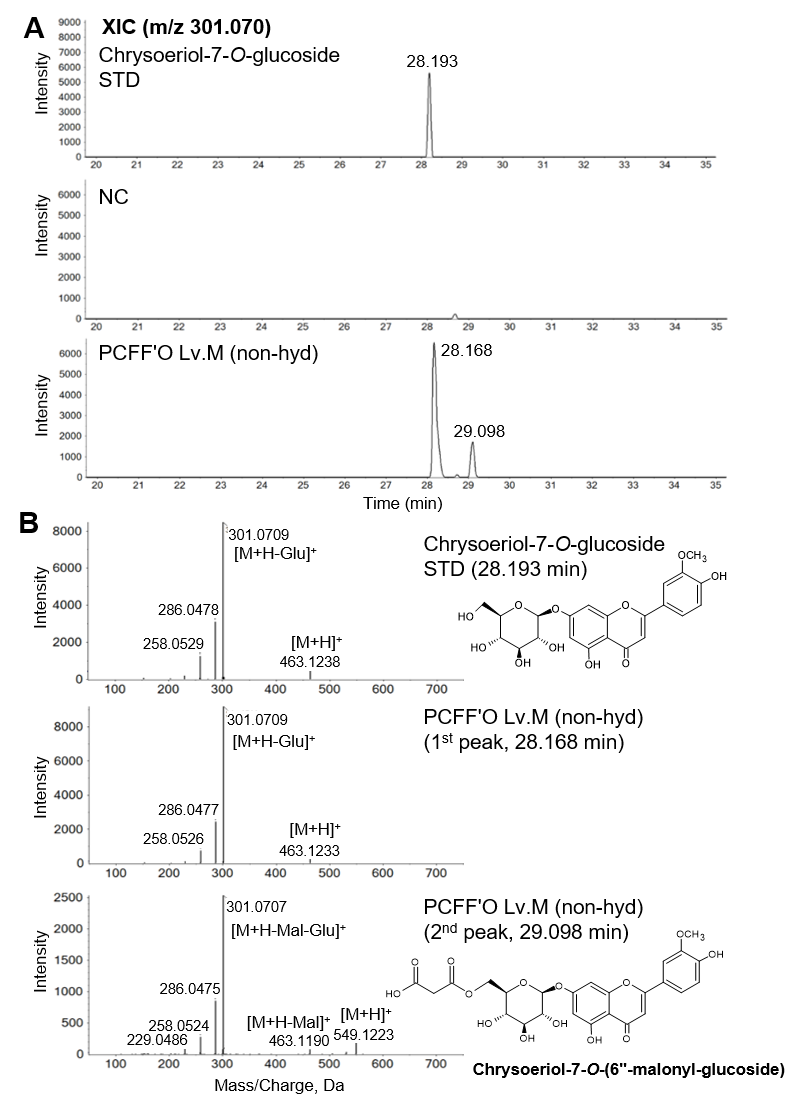


**Supplementary Figure 6.** Analysis of chrysoeriol glycoside accumulation in *N. benthamiana* leaves transfected with the PCFF'O level M vector. **(A)** Extracted-ion chromatogram (XIC) at m/z 301.07 representing protonated chrysoeriol aglycone in infiltrated *N. benthamiana* leaves. **(B)** MS/MS spectrum of the chrysoeriol-7-O-glucoside standard peak and two peaks detected in *N. benthamiana* leaves transfected with the PCFF'O level M vector. The first peak was identified as a chrysoeriol-7-O-glucoside and the second peak was expected as a chrysoeriol-7-O-(6''-malonyl-glucoside).


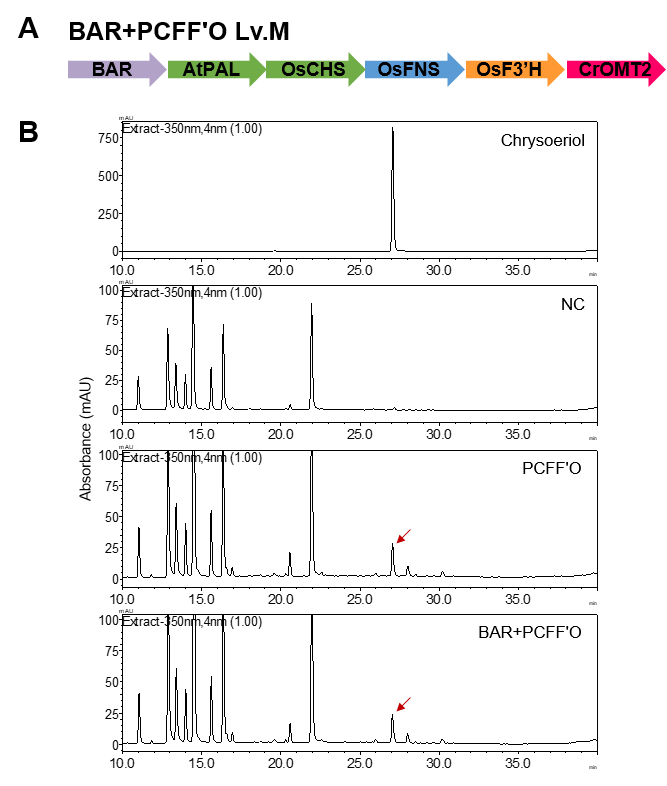


**Supplementary Figure 7.** Analysis of the activity of the multigene expression vector containing the basta-resistance gene (*BAR*) in chrysoeriol production by transient expression. **(A)** Schematic diagram of the multigene expression vector harboring the basta-resistance gene *BAR* (BAR+PCFF'O level M) for stable plant transformation. **(B)** HPLC chromatograms of chrysoeriol aglycone produced in *N. benthamiana* leaves infiltrated with infiltration medium (NC), the PCFF'O level M vector, or the BAR+PCFF'O level M vector. The chrysoeriol aglycone peak is indicated by a red arrow.


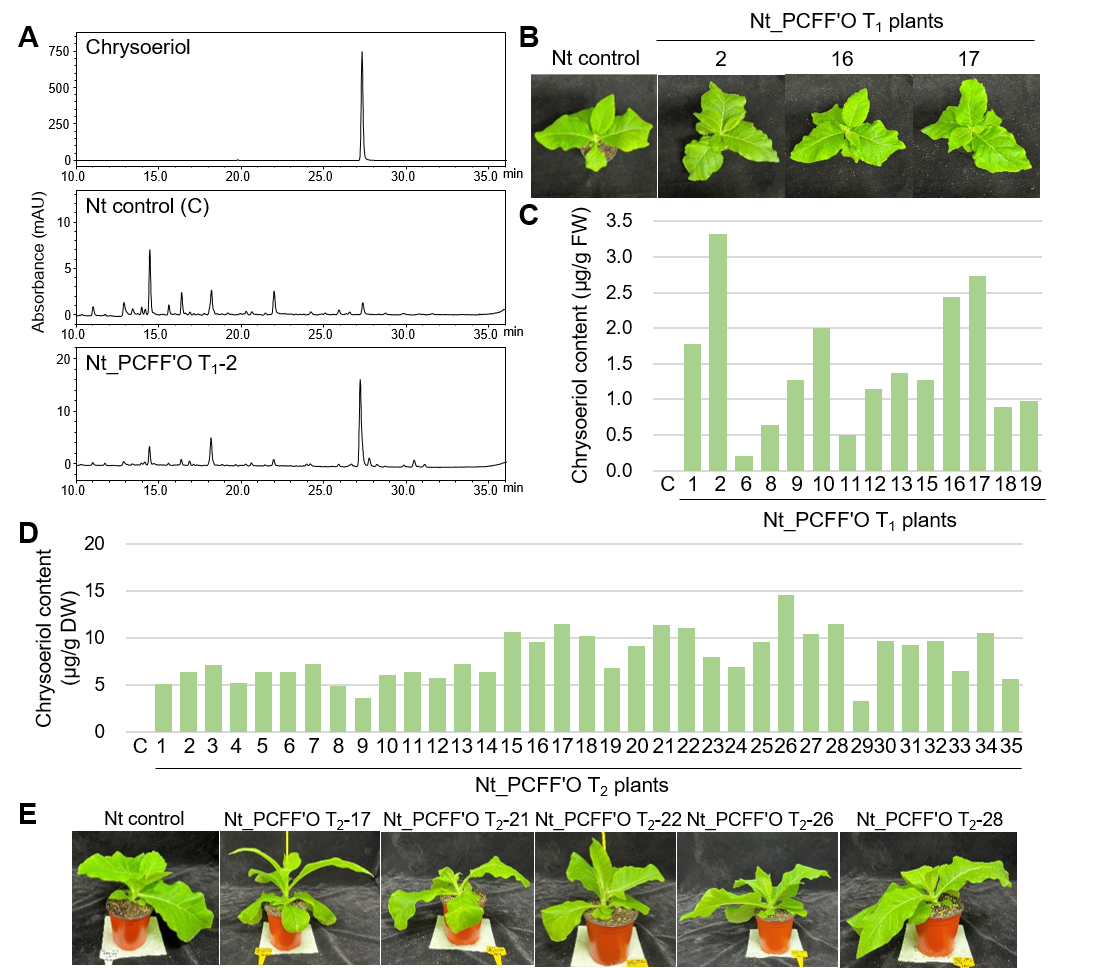


**Supplementary Figure 8.** Verification of chrysoeriol production in transgenic tobacco plants. Among the T_0_ *N. tabacum* transgenic plants, line T_0_-10, in which chrysoeriol was detected, was selected and T_1_ seeds were produced through self-fertilization. **(A to C)** Screening of T_1_ *N. tabacum* transgenic plants producing chrysoeriol by HPLC analysis. Line T_1_-2, with the highest chrysoeriol level, was selected to produce the T_2_ generation. C, non-transgenic plant. **(D)** HPLC analysis of chrysoeriol content in leaves of T_2_ generation transgenic plants. **(E)** Representative phenotypes of five T_2_ generation transgenic plants. Eight-week-old plants were photographed.


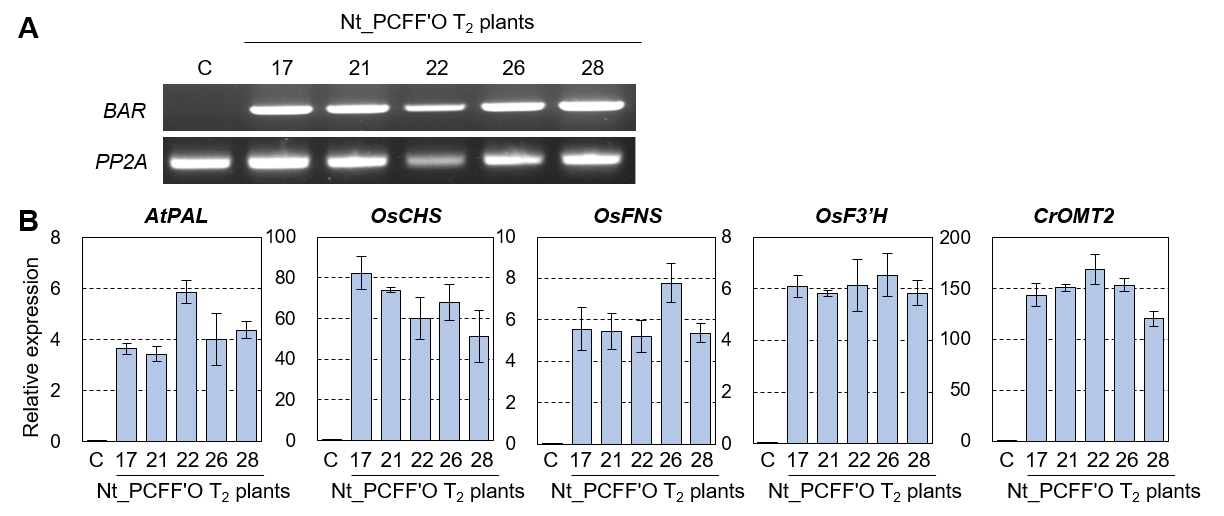


**Supplementary Figure 9.** Molecular characterization of transgenic plants. **(A)** PCR detection of the *BAR* gene in leaves from a non-transgenic plant (C) and T_2_ generation transgenic plant. **(B)** RT-qPCR analysis of the expression of five GOI in a non-transgenic plant and T_2_ generation transgenic plants. Genomic DNA and total RNA were isolated from the leaves of 6-week-old plants. Genomic DNA PCR and RT-qPCR were conducted using gene-specific primer sets. *PP2A* was used as an internal control. Bars indicate standard error (SE) of three technical replicates.


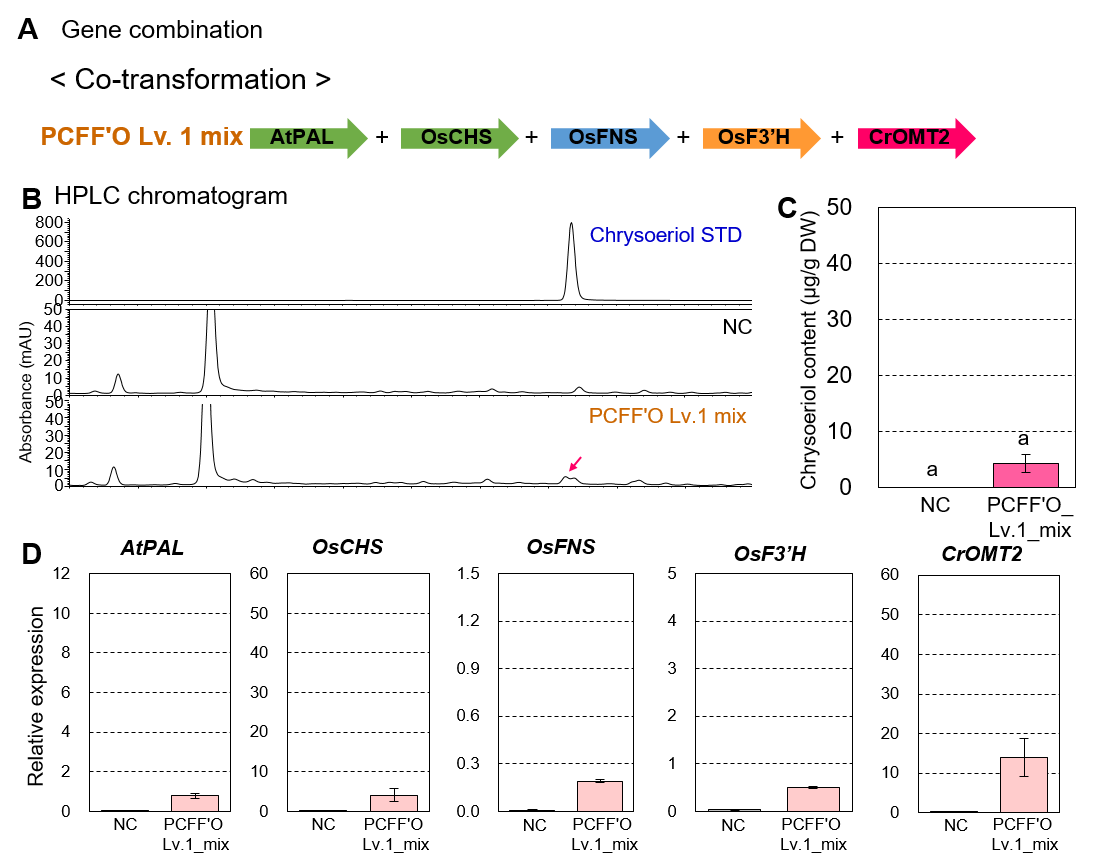


**Supplementary Figure 10.** Chrysoeriol production in *N. benthamiana* leaves via co-transformation. **(A)** Schematic diagram of co-transformation. During co-transformation, different *Agrobacterium* cultures harboring a single transcription unit were mixed at equal cell density and infiltrated into *N. benthamiana* leaves. **(B)** HPLC chromatograms of chrysoeriol produced in *N. benthamiana* leaves. Chrysoeriol was detected at 350 nm. The peak corresponding to chrysoeriol produced by transient expression is indicated by a red arrow. **(C)** Total content of chrysoeriol produced in *N. benthamiana* leaves transfected with infiltration medium (NC) or *Agrobacterium* cells harboring a mixture of individual transcription units. Bars indicate standard error (SE) of six technical replicates from two independent experiments. **(D)** Expression levels of five GOI following co-transformation. Total RNA was isolated from leaves at 6 days after infiltration (DAI), and RT-qPCR was conducted using gene-specific primer sets. *PP2A* was used as an internal control. Bars indicate standard error (SE) of three technical replicates.


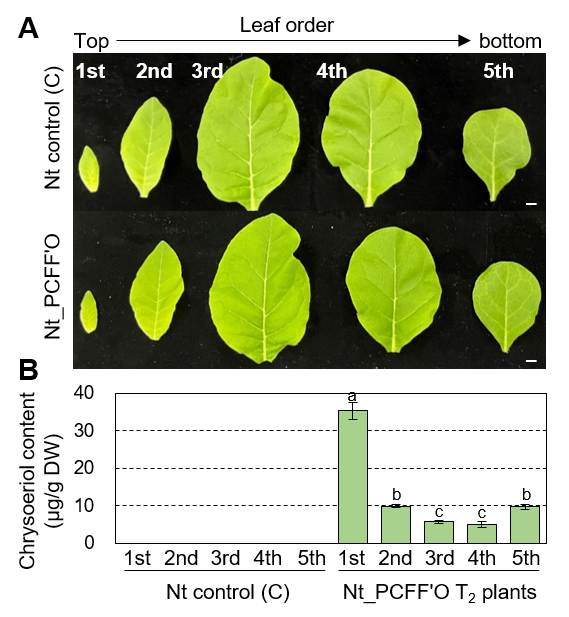


**Supplementary Figure 11.** Total content of chrysoeriol aglycone produced in leaves of *N. tabacum* transgenic plants by leaf order. **(A)** Leaves from non-transgenic plant (Nt control, C) and T_2_ generation transgenic plants expressing the five genes (Nt_PCFF'O T_2_ plants). Leaves detached from six-week-old plants were photographed. Leaf order indicates the leaf developmental age in order from the oldest leaf at the bottom to the youngest leaf at the top in a whole tobacco plant. Bars=1cm. **(B)** Total content of chrysoeriol aglycone produced in leaves of *N. tabacum* transgenic plants. Bars indicate standard error (SE) of technical replicates (n=4). Different letters represent significant differences based on ANOVA with Tukey HSD (*P* < 0.05).

## Supplementary Tables

**Supplementary Table 1.** Sequence alignment of the original sequences of the genes of interest with the codon optimized sequences.

| Genes  (Similarity) | Alignments with codon optimized sequences |
| --- | --- |
| OsCHS  (73%) | OsCHS ATGGCGGCGGCGGTGACGGTGGAGGAGGTGAGGAGGGCGCAGAGGGCGGAGGGGCCGGCG  OsCHS_Nta ATGGCTGCTGCAGTTACGGTGGAAGAAGTACGACGTGCTCAAAGAGCTGAGGGACCAGCA  ***** ** ** ** ******** ** ** * * ** ** ** ** ***** ** **  OsCHS ACGGTGCTGGCGATCGGGACGGCGACGCCGGCGAACTGCGTGTACCAGGCCGACTACCCG  OsCHS_Nta ACGGTTTTAGCCATCGGCACAGCCACTCCTGCTAATTGTGTTTATCAAGCAGACTATCCA  ***** * ** ***** ** ** ** ** ** ** ** ** ** ** ** ***** **  OsCHS GACTACTACTTCAGGATCACCAAGAGCGAGCACATGGTCGAGCTCAAGGAGAAGTTCAAG  OsCHS_Nta GATTATTACTTCAGAATAACAAAAAGCGAACACATGGTCGAACTCAAAGAAAAATTCAAA  ** ** ******** ** ** ** ***** *********** ***** ** ** *****  OsCHS AGGATGTGTGACAAGTCGCAGATCAGGAAGAGGTACATGCACCTGACGGAGGAGATCCTG  OsCHS_Nta AGGATGTGTGATAAGTCTCAGATAAGAAAAAGATACATGCATTTAACAGAGGAGATTTTG  *********** ***** ***** ** ** ** ******** * ** ******** **  OsCHS CAGGAGAACCCCAACATGTGCGCGTACATGGCGCCGTCGCTGGACGCGCGGCAGGACATC  OsCHS_Nta CAAGAAAATCCAAATATGTGCGCTTATATGGCACCTTCCTTAGATGCAAGGCAAGATATT  ** ** ** ** ** ******** ** ***** ** ** * ** ** **** ** **  OsCHS GTCGTCGTCGAGGTCCCCAAGCTGGGGAAGGCGGCGGCGCAGAAGGCGATCAAGGAGTGG  OsCHS_Nta GTTGTAGTTGAGGTGCCGAAGTTGGGGAAGGCTGCCGCTCAGAAAGCTATAAAGGAATGG  ** ** ** ***** ** *** ********** ** ** ***** ** ** ***** ***  OsCHS GGGCAGCCGCGCTCCCGCATCACCCACCTCGTCTTCTGCACCACCTCCGGCGTCGACATG  OsCHS_Nta GGACAGCCTAGGTCACGCATCACCCACCTAGTGTTTTGTACTACAAGTGGTGTAGATATG  ** ***** * ** ************** ** ** ** ** ** ** ** ** ***  OsCHS CCCGGCGCCGACTACCAGCTCGCCAAGATGCTCGGCCTGAGGCCCAACGTGAACCGCCTC  OsCHS_Nta CCAGGGGCTGATTACCAACTTGCAAAAATGCTCGGTCTAAGACCAAACGTGAACCGGTTG  ** ** ** ** ***** ** ** ** ******** ** ** ** *********** *  OsCHS ATGATGTACCAGCAGGGGTGCTTCGCCGGCGGCACGGTGCTCCGCGTCGCCAAGGACCTC  OsCHS_Nta ATGATGTATCAGCAGGGATGCTTTGCAGGAGGAACTGTTCTTCGCGTAGCTAAGGACTTG  ******** ******** ***** ** ** ** ** ** ** ***** ** ****** *  OsCHS GCCGAGAACAACCGCGGCGCGCGCGTCCTCGCCGTGTGCTCCGAGATCACGGCGGTGACG  OsCHS_Nta GCAGAGAATAATCGGGGTGCGCGAGTGCTGGCGGTTTGTTCGGAAATAACAGCAGTCACC  ** ***** ** ** ** ***** ** ** ** ** ** ** ** ** ** ** ** **  OsCHS TTCCGGGGGCCCTCCGAGTCCCACCTCGACTCCATGGTCGGGCAGGCGCTGTTCGGCGAC  OsCHS_Nta TTTCGAGGACCTTCAGAGTCTCACCTGGACTCCATGGTTGGTCAAGCTCTTTTTGGAGAT  ** ** ** ** ** ***** ***** *********** ** ** ** ** ** ** **  OsCHS GGCGCGGCGGCGGTGATCGTCGGCTCCGACCCCGACGAGGCCGTCGAGCGGCCGCTGTTC  OsCHS_Nta GGGGCAGCAGCTGTTATTGTGGGGTCTGATCCTGATGAGGCTGTAGAGAGGCCCTTGTTT  ** ** ** ** ** ** ** ** ** ** ** ** ***** ** *** **** ****  OsCHS CAGATGGTGTCGGCGAGCCAGACCATCCTCCCGGACAGCGAGGGCGCCATCGACGGCCAC  OsCHS_Nta CAAATGGTATCAGCAAGCCAGACTATCCTTCCGGACTCTGAGGGCGCAATTGATGGACAT  ** ***** ** ** ******** ***** ****** ******** ** ** ** **  OsCHS CTGAGGGAGGTCGGGCTGACGTTCCACCTGCTCAAGGACGTGCCGGGGCTCATCTCGAAG  OsCHS_Nta TTAAGAGAAGTTGGGCTGACATTTCATCTATTGAAAGATGTACCCGGGCTCATATCAAAG  * ** ** ** ******** ** ** ** * ** ** ** ** ******** ** ***  OsCHS AACATCGAGCGCGCGCTGGGCGACGCGTTCACACCGCTGGGGATCTCGGACTGGAACTCC  OsCHS_Nta AATATTGAAAGGGCCCTTGGTGATGCTTTCACTCCACTAGGGATTAGTGACTGGAATTCA  ** ** ** * ** ** ** ** ** ***** ** ** ***** ******** **  OsCHS ATCTTCTGGGTGGCGCACCCCGGAGGTCCGGCGATCCTGGACCAGGTGGAGGCGAAGGTT  OsCHS_Nta ATTTTTTGGGTTGCTCATCCTGGAGGTCCAGCTATTCTTGATCAAGTTGAAGCTAAGGTC  ** ** ***** ** ** ** ******** ** ** ** ** ** ** ** ** *****  OsCHS GGGCTGGACAAGGAGAGGATGAGGGCGACGCGCCACGTGCTGTCCGAGTACGGCAACATG  OsCHS_Nta GGCCTTGACAAAGAGAGAATGAGAGCAACACGTCATGTTTTGTCTGAATATGGAAACATG  ** ** ***** ***** ***** ** ** ** ** ** **** ** ** ** ******  OsCHS TCGAGCGCCTGCGTGCTCTTCATCCTCGACGAGATGCGCAAGCGCTCCGCCGAGGACGGC  OsCHS_Nta AGCAGTGCCTGCGTTCTGTTCATTCTGGATGAAATGAGGAAGCGTAGTGCAGAAGATGGT  ** ******** ** ***** ** ** ** *** * ***** ** ** ** **  OsCHS CACGCCACCACCGGCGAGGGCATGGACTGGGGCGTCCTCTTCGGCTTCGGCCCCGGCCTC  OsCHS_Nta CATGCTACCACTGGTGAAGGAATGGATTGGGGAGTGCTTTTCGGCTTTGGTCCAGGCTTG  ** ** ***** ** ** ** ***** ***** ** ** ******** ** ** *** *  OsCHS ACCGTTGAGACCGTCGTCCTCCACAGCGTCCCCATCACCGCCGGCGCCGCCGCCTGA  OsCHS_Nta ACCGTTGAGACTGTCGTCTTACATTCTGTGCCTATCACTGCTGGTGCCGCGGCATGA  *********** ****** * ** ** ** ***** ** ** ***** ** *** |
| OsFNS  (71%) | OsFNS ATGGCATCGTTGATGGAAGTGCAGGTGCCGCTGTTGGGAATGGGCACTACGATGGGCGCC  OsFNS_Nta ATGGCATCATTGATGGAGGTCCAAGTACCCCTACTCGGCATGGGTACAACAATGGGGGCT  ******** ******** ** ** ** ** ** * ** ***** ** ** ***** **  OsFNS CTGGCTCTGGCTCTCGTCGTCGTTGTGGTTGTGCACGTTGCCGTGAACGCCTTTGGGCGG  OsFNS_Nta TTGGCTCTAGCATTAGTCGTAGTAGTTGTGGTGCATGTTGCCGTGAATGCATTTGGGCGG  ******* ** * ***** ** ** ** ***** *********** ** *********  OsFNS CGGCGGCTTCCCCCGAGTCCGGCGAGCCTACCGGTGATCGGGCACCTTCACCTGCTCCGG  OsFNS_Nta CGCAGGTTACCGCCATCGCCGGCCAGCCTCCCGGTTATTGGCCACCTACATTTACTTAGG  ** ** * ** ** ***** ***** ***** ** ** ***** ** * ** **  OsFNS CCGCCGGTGCACCGCACCTTCCACGAGCTGGCGGCGAGGCTGGGCCCCCTGATGCACGTC  OsFNS_Nta CCTCCTGTTCACCGCACATTTCATGAACTTGCAGCACGGTTGGGACCTTTAATGCATGTA  ** ** ** ******** ** ** ** ** ** ** ** **** ** * ***** **  OsFNS CGGCTGGGGTCGACGCACTGCGTTGTGGCGAGCTCGGCGGAGGTGGCGGCGGAGCTGATC  OsFNS_Nta AGATTGGGCTCGACACATTGTGTGGTGGCATCCTCCGCAGAAGTTGCGGCAGAGCTCATC  * **** ***** ** ** ** ***** *** ** ** ** ***** ***** ***  OsFNS CGCAGCCACGAGGCGAAGATATCGGAGCGGCCGCTGACGGCGGTGGCCCGGCAGTTCGCG  OsFNS_Nta AGATCTCATGAGGCAAAAATATCAGAGAGGCCACTTACGGCTGTAGCAAGACAATTTGCG  * ** ***** ** ***** *** **** ** ***** ** ** * ** ** ***  OsFNS TACGAGTCGGCGGGGTTCGCGTTCGCGCCCTACAGCCCGCACTGGCGCTTCATGAAGCGG  OsFNS_Nta TATGAGTCTGCTGGTTTCGCTTTTGCTCCTTACAGTCCTCATTGGAGATTTATGAAGAGG  ** ***** ** ** ***** ** ** ** ***** ** ** *** * ** ****** **  OsFNS CTGTGCATGTCGGAGCTGCTGGGCCCGCGCACGGTGGAGCAGCTGCGCCCCGTGCGCCGA  OsFNS_Nta CTATGCATGAGTGAGCTACTTGGTCCAAGGACAGTTGAACAATTGCGACCTGTCAGAAGA  ** ****** ***** ** ** ** * ** ** ** ** **** ** ** * **  OsFNS GCCGGGCTGGTGTCGCTGCTGCGCCACGTGTTGTCGCAGCCGGAGGCGGAGGCGGTGGAC  OsFNS_Nta GCAGGTCTAGTCTCCCTTCTGCGACACGTGCTTTCACAGCCTGAAGCGGAGGCTGTTGAT  ** ** ** ** ** ** ***** ****** * ** ***** ** ******** ** **  OsFNS CTGACCCGCGAGCTCATCCGCATGTCCAACACCTCCATCATCCGCATGGCCGCCAGCACG  OsFNS_Nta TTAACACGTGAGTTGATTAGAATGTCTAACACTTCAATAATAAGGATGGCTGCTAGTACT  * ** ** *** * ** * ***** ***** ** ** ** * ***** ** ** **  OsFNS GTCCCCAGCAGCGTGACGGAGGAGGCGCAGGAGCTGGTGAAGGTGGTGGCGGAGCTGGTG  OsFNS_Nta GTACCAAGTTCTGTTACTGAGGAAGCTCAAGAATTAGTGAAGGTCGTGGCCGAATTGGTT  ** ** ** ** ** ***** ** ** ** * ******** ***** ** ****  OsFNS GGCGCCTTCAACGCCGACGACTACATCGCCCTGTGCCGTGGCTGGGACCTGCAGGGCCTC  OsFNS_Nta GGGGCATTCAATGCTGATGATTATATTGCACTATGTAGAGGATGGGATTTACAAGGATTG  ** ** ***** ** ** ** ** ** ** ** ** * ** ***** * ** ** *  OsFNS GGGCGCCGTGCGGCCGACGTGCACAAGAGGTTCGACGCGCTGCTGGAGGAGATGATCAGG  OsFNS_Nta GGCCGAAGGGCTGCGGACGTTCATAAAAGATTCGACGCCTTGTTAGAAGAAATGATTCGA  ** ** * ** ** ***** ** ** ** ******** ** * ** ** ***** *  OsFNS CACAAGGAGGAGGCTAGGATGCGGAAGAAGACAGACACAGACGTCGGCAGCAAGGACCTG  OsFNS_Nta CACAAGGAGGAGGCTCGTATGAGGAAGAAAACTGATACAGATGTAGGAAGCAAAGATCTT  *************** * *** ******* ** ** ***** ** ** ***** ** **  OsFNS CTTGACATCCTGCTGGACAAGGCGGAGGACGGCGCGGCGGAGGTGAAGCTCACCAGAGAC  OsFNS_Nta CTTGATATACTTTTAGACAAGGCTGAAGATGGAGCTGCGGAGGTAAAGTTGACCCGGGAC  ***** ** ** * ******** ** ** ** ** ******** *** * *** * ***  OsFNS AACATCAAGGCCTTCATCATCGACGTTGTTACTGCCGGGTCCGACACTTCGGCCGCCATG  OsFNS_Nta AACATCAAAGCTTTCATTATAGATGTCGTCACTGCTGGATCTGACACCTCAGCCGCAATG  ******** ** ***** ** ** ** ** ***** ** ** ***** ** ***** ***  OsFNS GTGGAGTGGATGGTGGCGGAGCTGATGAACCACCCGGAGGCCCTGCGCAAGGTGCGGGAG  OsFNS_Nta GTGGAATGGATGGTTGCTGAACTGATGAATCATCCAGAAGCTCTTAGAAAAGTTAGAGAG  ***** ******** ** ** ******** ** ** ** ** ** * ** ** * ***  OsFNS GAGATCGAGGCGGTGGTGGGGCGGGACAGGATCGCCGGCGAGGGGGACCTGCCGAGACTG  OsFNS_Nta GAAATTGAGGCGGTAGTTGGTCGTGACAGAATAGCTGGGGAAGGTGATCTGCCTCGTCTG  ** ** ******** ** ** ** ***** ** ** ** ** ** ** ***** * ***  OsFNS CCGTATCTGCAGGCGGCGTACAAGGAGACGCTGCGGTTGAGGCCGGCGGCGCCGATCGCG  OsFNS_Nta CCTTATTTGCAGGCTGCCTACAAGGAAACCCTTAGGTTACGGCCAGCAGCTCCTATTGCT  ** *** ******* ** ******** ** ** **** **** ** ** ** ** **  OsFNS CACAGGCAGTCGACGGAGGAGATCCAGATCCGAGGGTTCAGGGTGCCGGCGCAGACGGCG  OsFNS_Nta CACAGGCAGTCAACTGAAGAGATTCAAATTCGTGGTTTCAGAGTTCCAGCTCAGACTGCA  *********** ** ** ***** ** ** ** ** ***** ** ** ** ***** **  OsFNS GTGTTCATCAACGTGTGGGCCATCGGGCGAGACCCGGCGTACTGGGAGGAGCCGCTGGAG  OsFNS_Nta GTTTTCATCAATGTTTGGGCTATCGGACGTGATCCTGCTTATTGGGAAGAACCATTGGAA  ** ******** ** ***** ***** ** ** ** ** ** ***** ** ** ****  OsFNS TTCAGGCCGGAGCGGTTCCTCGCCGGCGGCGGCGGCGAGGGCGTGGAGCCGCGCGGGCAG  OsFNS_Nta TTTCGACCCGAGAGGTTTCTGGCCGGGGGAGGTGGAGAAGGCGTTGAGCCAAGAGGGCAA  ** * ** *** **** ** ***** ** ** ** ** ***** ***** * *****  OsFNS CACTTCCAGTTCATGCCGTTCGGGAGCGGCCGGCGCGGGTGCCCCGGGATGGGGCTTGCG  OsFNS_Nta CATTTTCAGTTCATGCCATTTGGATCTGGAAGGAGAGGTTGCCCTGGAATGGGATTGGCA  ** ** *********** ** ** ** ** * ** ***** ** ***** * **  OsFNS CTGCAGTCGGTGCCGGCGGTGGTGGCGGCGCTGCTGCAGTGCTTCGATTGGCAGTGCATG  OsFNS_Nta CTGCAGAGTGTTCCCGCCGTCGTTGCAGCCTTACTCCAATGCTTTGATTGGCAATGTATG  ****** ** ** ** ** ** ** ** * ** ** ***** ******** ** ***  OsFNS GACAATAAGTTGATAGACATGGAGGAGGCAGACGGCCTGGTTTGCGCTCGGAAGCATCGC  OsFNS_Nta GATAATAAACTTATTGATATGGAAGAAGCAGATGGTTTGGTGTGTGCTAGGAAACATAGA  ** ***** * ** ** ***** ** ***** ** **** ** *** **** *** *  OsFNS CTCCTCCTCCACGCCCACCCGCGCCTCCACCCTTTCCCGCCGCTCCTCTAG  OsFNS_Nta CTCTTGCTTCATGCACATCCAAGACTTCATCCATTTCCACCTCTCCTGTGA  *** * ** ** ** ** ** * ** ** ** ** ** ** ***** * |
| ZmFNSI  (70%) | ZmFNSI ATGGCGGAGCACCTCCTGTCGACGGCCGTGCACGACACGCTGCCGGGGAGCTACGTGCGG  ZmFNSI_Nta ATGGCCGAGCATCTCCTCTCCACTGCTGTCCATGATACCTTACCTGGATCGTATGTGAGG  ***** ***** ***** ** ** ** ** ** ** ** * ** ** ** *** **  ZmFNSI CCGGAGCCGGAGCGCCCGCGCCTCGCGGAGGTCGTGACCGGCGCGCGCATCCCCGTCGTG  ZmFNSI_Nta CCAGAGCCTGAGAGGCCGAGACTCGCTGAAGTGGTGACTGGTGCAAGGATTCCTGTTGTA  ** ***** *** * *** * ***** ** ** ***** ** ** * ** ** ** **  ZmFNSI GACCTGGGCAGCCCCGACCGCGGCGCGGTCGTGGCCGCCGTCGGCGACGCCTGCCGCTCG  ZmFNSI_Nta GATCTGGGTTCTCCCGACCGGGGTGCTGTGGTTGCCGCGGTTGGTGATGCTTGCAGATCT  ** ***** ******** ** ** ** ** ***** ** ** ** ** *** * **  ZmFNSI CACGGCTTCTTCCAGGTCGTCAACCACGGGATACACGCCGCCCTGGTCGCGGCGGTGATG  ZmFNSI_Nta CATGGATTTTTTCAGGTTGTAAATCACGGGATTCATGCAGCCTTGGTTGCAGCTGTAATG  ** ** ** ** ***** ** ** ******** ** ** *** **** ** ** ** ***  ZmFNSI GCCGCGGGGCGCGGCTTCTTCCGGCTGCCCCCCGAGGAGAAGGCCAAGCTCTACTCCGAC  ZmFNSI_Nta GCTGCTGGCCGTGGTTTCTTCAGGTTGCCTCCTGAAGAGAAAGCAAAGCTGTACTCTGAT  ** ** ** ** ** ****** ** **** ** ** ***** ** ***** ***** **  ZmFNSI GACCCCGCCAGGAAGATCCGGCTGTCCACCAGCTTCAACGTGCGCAAGGAGACGGTGCAC  ZmFNSI_Nta GATCCTGCTAGGAAGATCCGGTTAAGCACATCATTCAATGTTAGGAAGGAGACAGTTCAC  ** ** ** ************ * *** ***** ** * ******** ** ***  ZmFNSI AACTGGCGCGACTACCTCCGCCTGCACTGCCATCCCCTCGACGAGTTCCTGCCCGATTGG  ZmFNSI_Nta AATTGGAGAGATTATCTTCGATTACACTGTCATCCACTGGACGAATTTCTTCCTGATTGG  ** *** * ** ** ** ** * ***** ***** ** ***** ** ** ** ******  ZmFNSI CCGTCCAACCCGCCCGATTTCAAGGAGACCATGGGCACCTACTGCAAGGAGGTCCGGGAG  ZmFNSI_Nta CCAAGTAACCCTCCAGACTTCAAGGAAACAATGGGAACTTACTGCAAAGAAGTTAGAGAG  ** ***** ** ** ******** ** ***** ** ******** ** ** * ***  ZmFNSI CTCGGGTTCAGGCTGTACGCCGCGATCTCGGAGAGCCTGGGCCTAGAGGCGAGCTACATG  ZmFNSI_Nta CTAGGCTTTCGCCTCTATGCCGCAATTTCTGAATCACTTGGGCTAGAAGCTTCTTACATG  ** ** ** * ** ** ***** ** ** ** ** ** ***** ** ******  ZmFNSI AAGGAAGCGCTGGGGGAGCAGGAGCAGCACATGGCGGTCAACTTCTACCCGCCGTGCCCG  ZmFNSI_Nta AAAGAGGCATTGGGAGAACAAGAACAGCATATGGCGGTGAACTTCTATCCACCCTGTCCA  ** ** ** **** ** ** ** ***** ******** ******** ** ** ** **  ZmFNSI GAGCCGGAGCTCACCTACGGCCTCCCGGCGCACACCGACCCCAACGCGCTCACCATCCTG  ZmFNSI_Nta GAGCCAGAATTGACCTATGGACTACCAGCGCACACTGATCCAAATGCTCTCACGATATTG  ***** ** * ***** ** ** ** ******** ** ** ** ** ***** ** **  ZmFNSI CTCATGGACCCGGACGTCGCCGGCCTGCAGGTGCTCCACGCCGGCCAGTGGGTCGCCGTC  ZmFNSI_Nta TTGATGGATCCCGATGTTGCCGGCTTACAGGTCCTTCATGCTGGGCAATGGGTGGCAGTC  * ***** ** ** ** ****** * ***** ** ** ** ** ** ***** ** ***  ZmFNSI AACCCGCAGCCCGGCGCGCTCATCATCAACATCGGCGACCAGCTGCAGGCGCTGAGCAAC  ZmFNSI_Nta AACCCACAACCTGGGGCACTTATAATCAATATTGGAGATCAACTTCAAGCTCTGTCAAAT  ***** ** ** ** ** ** ** ***** ** ** ** ** ** ** ** *** **  ZmFNSI GGGCAGTACCGGAGCGTGTGGCACCGCGCGGTGGTGAACTCGGACCGGGAGCGCATGTCC  ZmFNSI_Nta GGTCAATATCGTTCCGTTTGGCATAGAGCAGTAGTCAACAGCGACAGAGAAAGAATGAGT  ** ** ** ** *** ***** * ** ** ** *** *** * ** * ***  ZmFNSI GTGGCGTCGTTCCTGTGCCCGTGCAACCACGTCGTGCTCGGCCCCGCGCGGAAGCTCGTC  ZmFNSI_Nta GTAGCTTCATTTTTATGTCCTTGCAATCATGTTGTGCTAGGACCGGCAAGAAAGCTTGTC  ** ** ** ** * ** ** ***** ** ** ***** ** ** ** * ***** ***  ZmFNSI ACCGAGGACACCCCGGCCGTGTACAGGAACTACACGTACGACAAGTACTACGCCAAGTTC  ZmFNSI_Nta ACAGAGGATACTCCGGCTGTTTACAGAAATTACACATATGATAAATATTATGCAAAATTT  ** ***** ** ***** ** ***** ** ***** ** ** ** ** ** ** ** **  ZmFNSI TGGAGCAGGAACCTGGACCAGGAGCACTGCCTCGAGCTCTTCAGAACCTAG  ZmFNSI_Nta TGGAGTCGAAATCTTGACCAGGAACATTGTTTGGAGTTGTTTCGTACCTGA  ***** * ** ** ******** ** ** * *** * ** * **** |
| OsF3'H  (73%) | OsF3'H ATGGACGTTGTGCCTCTCCCGCTGCTGCTCGGCTCCCTGGCCGTGTCCGCCGCCGTGTGG  OsF3'H_Nta ATGGACGTAGTGCCATTACCCCTTCTGCTTGGATCTCTGGCCGTTTCTGCGGCTGTCTGG  ******** ***** * ** ** ***** ** ** ******** ** ** ** ** ***  OsF3'H TACCTTGTGTACTTCCTCCGCGGCGGCAGCGGCGGCGACGCGGCGAGGAAGCGGCGGCCT  OsF3'H_Nta TATCTTGTCTATTTCCTGAGAGGAGGATCGGGTGGTGATGCTGCAAGGAAACGTCGACCT  ** ***** ** ***** * ** ** ** ** ** ** ** ***** ** ** ***  OsF3'H TTGCCACCCGGGCCACGCGGGTGGCCCGTGCTGGGCAACCTGCCGCAGCTCGGCGACAAG  OsF3'H_Nta CTACCACCTGGTCCAAGGGGCTGGCCTGTATTGGGAAACTTGCCACAGTTGGGAGACAAA  * ***** ** *** * ** ***** ** **** *** **** *** * ** *****  OsF3'H CCGCACCACACCATGTGCGCCCTGGCGCGGCAGTACGGCCCGCTGTTCCGGCTCCGGTTC  OsF3'H_Nta CCTCACCATACAATGTGTGCACTTGCACGGCAATATGGACCGTTGTTCAGGCTGCGTTTT  ** ***** ** ***** ** ** ** ***** ** ** *** ***** **** ** **  OsF3'H GGCTGCGCCGAGGTGGTGGTGGCCGCGTCGGCGCCCGTGGCTGCGCAGTTCCTGCGCGGG  OsF3'H_Nta GGATGCGCTGAAGTAGTTGTCGCAGCTAGCGCACCTGTTGCTGCGCAGTTTTTACGCGGT  ** ***** ** ** ** ** ** ** ** ** ** *********** * *****  OsF3'H CACGATGCCAACTTCAGCAACCGCCCGCCCAACTCGGGCGCCGAGCACGTCGCGTACAAC  OsF3'H_Nta CATGATGCTAATTTTTCAAACAGACCTCCTAATAGTGGCGCAGAGCACGTAGCTTACAAT  ** ***** ** ** *** * ** ** ** ***** ******** ** *****  OsF3'H TACCAGGACCTCGTCTTCGCGCCCTACGGTGCTCGCTGGCGCGCCCTGCGGAAGCTGTGC  OsF3'H_Nta TATCAAGATCTGGTCTTTGCACCTTACGGTGCGCGGTGGAGAGCTTTAAGAAAGCTCTGT  ** ** ** ** ***** ** ** ******** ** *** * ** * * ***** **  OsF3'H GCGCTCCACCTCTTCTCGGCCAAGGCGCTCGACGACCTCCGAGCAGTCCGGGAGGGCGAG  OsF3'H_Nta GCTCTTCACCTTTTCTCTGCCAAGGCATTGGATGACTTGCGTGCTGTGAGAGAAGGAGAG  ** ** ***** ***** ******** * ** *** * ** ** ** * ** ** ***  OsF3'H GTCGCGCTCATGGTGAGGAACCTCGCTCGGCAGCAGGCGGCGTCAGTGGCGCTGGGGCAG  OsF3'H_Nta GTCGCTCTGATGGTGAGAAACTTAGCCAGGCAACAAGCCGCTAGTGTTGCACTTGGACAA  ***** ** ******** *** * ** **** ** ** ** ** ** ** ** **  OsF3'H GAAGCGAACGTCTGCGCCACGAACACGCTGGCCCGCGCCACCATCGGTCACCGGGTGTTC  OsF3'H_Nta GAGGCAAATGTTTGCGCCACAAATACCTTAGCAAGAGCAACGATTGGGCACAGGGTATTT  ** ** ** ** ******** ** ** * ** * ** ** ** ** *** **** **  OsF3'H GCCGTCGACGGCGGGGAAGGCGCAAGGGAGTTCAAGGAGATGGTTGTGGAGCTGATGCAG  OsF3'H_Nta GCCGTGGATGGAGGAGAAGGGGCGCGTGAATTTAAAGAGATGGTGGTTGAGCTAATGCAG  ***** ** ** ** ***** ** * ** ** ** ******** ** ***** ******  OsF3'H CTCGCCGGCGTTTTCAACGTCGGGGACTTCGTGCCGGCGCTCCGGTGGCTCGACCCGCAG  OsF3'H_Nta TTAGCAGGAGTATTCAATGTCGGAGACTTCGTTCCGGCACTGAGATGGCTAGACCCACAA  * ** ** ** ***** ***** ******** ***** ** * ***** ***** **  OsF3'H GGCGTCGTGGCAAAGATGAAGAGGCTGCACCGTCGGTACGACAACATGATGAACGGATTC  OsF3'H_Nta GGCGTTGTTGCAAAAATGAAGAGGTTGCATAGGAGATATGATAATATGATGAATGGTTTC  ***** ** ***** ********* **** * * ** ** ** ******** ** ***  OsF3'H ATCAACGAAAGGAAGGCCGGGGCGCAGCCCGACGGGGTCGCCGCTGGCGAGCACGGCAAC  OsF3'H_Nta ATCAATGAAAGAAAAGCTGGTGCTCAGCCCGATGGAGTAGCGGCCGGCGAACATGGAAAT  ***** ***** ** ** ** ** ******** ** ** ** ** ***** ** ** **  OsF3'H GACCTTCTAAGCGTGCTGCTGGCGAGGATGCAGGAGGAGCAGAAGCTGGACGGCGACGGC  OsF3'H_Nta GATTTACTCTCTGTTCTTCTCGCTCGCATGCAAGAAGAGCAGAAGTTGGACGGTGACGGG  ** * ** ** ** ** ** * ***** ** ********* ******* *****  OsF3'H GAAAAGATCACCGAAACTGACATCAAAGCTCTGCTCCTGAACCTATTCACTGCGGGGACG  OsF3'H_Nta GAGAAGATCACAGAAACCGATATTAAGGCTCTACTTCTCAACCTGTTTACTGCTGGGACA  ** ******** ***** ** ** ** ***** ** ** ***** ** ***** *****  OsF3'H GATACGACATCGAGCACGGTGGAGTGGGCACTGGCGGAGCTGATCCGGCACCCGGACGTC  OsF3'H_Nta GATACGACCTCATCGACTGTTGAATGGGCACTAGCGGAGTTGATTAGACATCCAGATGTT  ******** ** ** ** ** ******** ****** **** * ** ** ** **  OsF3'H CTCAAGGAGGCCCAGCATGAGCTTGACACCGTCGTCGGTAGGGGTCGTCTCGTGTCCGAG  OsF3'H_Nta TTGAAAGAAGCTCAACATGAACTTGATACTGTTGTTGGCCGTGGGAGATTAGTGTCTGAA  * ** ** ** ** ***** ***** ** ** ** ** * ** * * ***** **  OsF3'H TCTGACCTTCCACGCCTCCCCTACCTCACCGCGGTGATCAAGGAGACGTTTCGGCTTCAC  OsF3'H_Nta AGCGATCTCCCTCGTCTCCCATACCTTACAGCAGTAATAAAGGAAACATTTAGGTTACAT  ** ** ** ** ***** ***** ** ** ** ** ***** ** *** ** * **  OsF3'H CCGTCAACGCCGCTCTCACTGCCTCGGGAGGCTGCAGAGGAGTGTGAGGTGGACGGCTAC  OsF3'H_Nta CCGTCCACCCCGCTGTCACTCCCCCGAGAGGCAGCTGAGGAGTGTGAAGTCGATGGTTAC  ***** ** ***** ***** ** ** ***** ** *********** ** ** ** ***  OsF3'H CGTATCCCCAAGGGCGCTACCCTCCTAGTCAACGTCTGGGCTATAGCCCGTGACCCGACC  OsF3'H_Nta CGCATTCCAAAAGGCGCTACTCTCCTTGTAAATGTGTGGGCAATTGCTAGGGATCCAACT  ** ** ** ** ******** ***** ** ** ** ***** ** ** * ** ** **  OsF3'H CAATGGCCCGACCCGCTACAGTACCAGCCTTCTCGGTTTCTCCCCGGCAGGATGCATGCA  OsF3'H_Nta CAATGGCCAGATCCTCTTCAGTATCAGCCTTCAAGATTCTTGCCAGGTAGGATGCATGCT  ******** ** ** ** ***** ******** * ** * ** ** ***********  OsF3'H GACGTGGATGTCAAGGGTGCTGATTTCGGCCTGATACCATTCGGAGCAGGACGGAGAATA  OsF3'H_Nta GATGTCGACGTGAAAGGTGCAGATTTTGGGCTTATACCTTTTGGTGCTGGTCGGCGAATA  ** ** ** ** ** ***** ***** ** ** ***** ** ** ** ** *** *****  OsF3'H TGCGCTGGCCTTAGTTGGGGCTTGCGGATGGTCACACTGATGACTGCCACGCTAGTGCAC  OsF3'H_Nta TGTGCTGGGCTAAGTTGGGGTTTAAGAATGGTGACACTAATGACTGCCACTCTTGTTCAT  ** ***** ** ******** ** * ***** ***** *********** ** ** **  OsF3'H GGGTTCGACTGGACCTTGGCTAACGGCGCGACTCCGGACAAGCTCAACATGGAGGAGGCC  OsF3'H_Nta GGCTTTGATTGGACTTTGGCTAACGGAGCAACACCTGATAAGCTCAACATGGAAGAAGCA  ** ** ** ***** *********** ** ** ** ** ************** ** **  OsF3'H TATGGGCTCACCTTGCAGAGGGCCGTGCCGTTGATGGTCCAGCCCGTGCCAAGGCTGCTT  OsF3'H_Nta TATGGCTTGACTCTACAGAGGGCAGTTCCCTTGATGGTTCAACCAGTGCCACGGTTGTTA  ***** * ** * ******** ** ** ******** ** ** ****** ** ** *  OsF3'H CCATCGGCTTATGGAGTATAA  OsF3'H_Nta CCGTCCGCTTATGGTGTTTGA  ** ** ******** ** * * |
| ROMT9  (74%) | ROMT9 ATGGTGGACCGCATGCTCCGCCTGCTCGCCTCCTACAACGTCGTCAGGTGCGAGATGGAG  ROMT9_Nta ATGGTGGATAGAATGTTACGCTTGTTGGCCTCTTACAATGTTGTAAGGTGTGAAATGGAA  ******** * *** * *** ** * ***** ***** ** ** ***** ** *****  ROMT9 GAGGGCGCCGACGGCAAGCTCTCCCGCCGCTACGCCGCCGCGCCGGTGTGCAAGTGGCTG  ROMT9_Nta GAAGGTGCTGATGGCAAACTTAGCAGGCGGTATGCTGCGGCACCAGTCTGCAAGTGGCTG  ** ** ** ** ***** ** * * ** ** ** ** ** ** ** ************  ROMT9 ACGCCCAACGAGGACGGCGTCTCCATGGCCGCCCTCGCCCTCATGAACCAGGACAAGGTC  ROMT9_Nta ACTCCAAATGAAGATGGAGTTTCAATGGCTGCCCTAGCCTTGATGAATCAGGATAAGGTG  ** ** ** ** ** ** ** ** ***** ***** *** * ***** ***** *****  ROMT9 CTCATGGAGAGCTGGTACTACCTTAAGGACGCAGTCCTGGACGGCGGCATCCCGTTCAAC  ROMT9_Nta CTCATGGAGAGTTGGTATTACCTGAAAGATGCAGTTCTTGATGGTGGCATCCCCTTCAAC  *********** ***** ***** ** ** ***** ** ** ** ******** ******  ROMT9 AAGGCGTACGGGATGACGGCGTTCGAGTACCACGGCACGGACGCCCGCTTCAACCGCGTC  ROMT9_Nta AAAGCATATGGGATGACAGCTTTTGAATATCACGGTACTGATGCAAGGTTTAACAGAGTT  ** ** ** ******** ** ** ** ** ***** ** ** ** * ** *** * **  ROMT9 TTCAACGAGGGCATGAAGAACCACTCCGTCATCATCACCAAGAAGCTGCTCGACCTCTAC  ROMT9_Nta TTCAACGAGGGAATGAAGAACCATTCAGTTATTATCACAAAGAAGCTATTAGACCTCTAC  *********** *********** ** ** ** ***** ******** * *********  ROMT9 ACCGGCTTCGACGCCGCCTCCACCGTCGTCGACGTCGGCGGCGGCGTGGGCGCCACTGTG  ROMT9_Nta ACAGGCTTTGATGCTGCATCCACCGTGGTAGATGTTGGAGGCGGAGTGGGAGCTACAGTG  ** ***** ** ** ** ******** ** ** ** ** ***** ***** ** ** ***  ROMT9 GCCGCCGTCGTCTCCCGCCACCCGCACATCCGGGGGATCAACTACGACCTCCCCCACGTC  ROMT9_Nta GCTGCTGTTGTCAGTCGACACCCACATATACGTGGAATCAATTATGACCTTCCTCACGTA  ** ** ** *** ** ***** ** ** ** ** ***** ** ***** ** *****  ROMT9 ATCTCCGAGGCGCCGCCGTTCCCCGGGGTGGAGCACGTCGGCGGCGACATGTTCGCCTCC  ROMT9_Nta ATTTCAGAAGCACCACCTTTTCCTGGTGTGGAGCATGTGGGGGGAGACATGTTTGCTTCT  ** ** ** ** ** ** ** ** ** ******** ** ** ** ******** ** **  ROMT9 GTGCCCCGCGGCGGCGACGCCATCCTGATGAAGTGGATCCTCCACGACTGGAGCGACGAG  ROMT9_Nta GTCCCCCGTGGTGGTGACGCCATTCTTATGAAATGGATTCTGCATGATTGGTCTGATGAA  ** ***** ** ** ******** ** ***** ***** ** ** ** *** ** **  ROMT9 CACTGCGCGCGGCTGCTCAAGAACTGCTACGACGCGCTGCCGGAGCACGGGAAGGTGGTG  ROMT9_Nta CATTGTGCTCGACTTCTCAAAAATTGCTATGATGCCTTGCCTGAGCACGGGAAAGTTGTA  ** ** ** ** ** ***** ** ***** ** ** **** *********** ** **  ROMT9 GTGGTGGAGTGCGTGCTGCCGGAGAGCTCCGACGCGACGGCGAGGGAGCAGGGGGTGTTC  ROMT9_Nta GTAGTTGAATGTGTTCTTCCGGAATCTTCTGACGCGACGGCAAGAGAACAAGGTGTTTTT  ** ** ** ** ** ** ***** ** *********** ** ** ** ** ** **  ROMT9 CACGTCGACATGATCATGCTCGCCCACAACCCCGGCGGCAAGGAGAGGTACGAGAGGGAG  ROMT9_Nta CATGTTGATATGATAATGTTGGCTCATAATCCTGGAGGGAAGGAAAGGTATGAGAGAGAG  ** ** ** ***** *** * ** ** ** ** ** ** ***** ***** ***** ***  ROMT9 TTCAGGGAGCTCGCCCGCGCCGCCGGATTCACCGGCTTCAAGGCCACCTACATCTACGCC  ROMT9_Nta TTCAGAGAGTTAGCAAGAGCAGCTGGATTCACTGGTTTCAAGGCCACTTACATATATGCA  ***** *** * ** * ** ** ******** ** *********** ***** ** **  ROMT9 AACGCCTGGGCCATCGAGTTCACCAAGTAG  ROMT9_Nta AATGCTTGGGCAATTGAATTTACCAAATGA  ** ** ***** ** ** ** ***** * |
| CrOMT2  (76%) | CrOMT2 ATGGGTTCAACCAGTTCAGAAACTCAAATAAGTCCAGCCCAAGGCTCGGATGAAGAGGCA  CrOMT2_Nta ATGGGAAGCACTTCCTCCGAAACTCAGATATCACCTGCTCAAGGATCAGATGAGGAAGCA  ***** ** ** ******** *** ** ** ***** ** ***** ** ***  CrOMT2 AACCTCTTGGCCATGCAATTAACCAGTGCCTCAGTCTTGCCTATGGTTCTCAAATCAGCC  CrOMT2_Nta AATTTGCTAGCAATGCAACTGACATCTGCTTCAGTGCTTCCTATGGTTCTCAAGTCTGCA  ** * * ** ****** * ** *** ***** * ************** ** **  CrOMT2 ATTGAGCTTGATCTTTTAGAGATCATCGCTAAAGCTGGGCCAGATGCTTTCATGTCTCCA  CrOMT2_Nta ATTGAACTCGATCTACTAGAGATTATTGCAAAAGCAGGGCCAGATGCATTCATGTCTCCA  ***** ** ***** ******* ** ** ***** *********** ************  CrOMT2 AAAGACATAGCTTCTCAGCTGCCCACAAAGAACCCAGATGCCCATATCGTGCTTGATCGT  CrOMT2_Nta AAGGATATAGCATCTCAGTTGCCAACAAAGAACCCCGACGCTCATATTGTATTAGACCGT  ** ** ***** ****** **** *********** ** ** ***** ** * ** ***  CrOMT2 ATATTGCGCCTTCTGGCGAGCTATTCAGTCCTTAATTGCTCTTTGCGCAATCTCCCCGAC  CrOMT2_Nta ATCCTTCGACTTCTCGCGAGTTACAGTGTCTTAAACTGTTCATTAAGAAATCTGCCTGAT  ** * ** ***** ***** ** *** * ** ** ** ** * ***** ** **  CrOMT2 GGCAAAGTTGAGAGGCTTTATGGCCTTGCCCCCGTTTGTAAATTCCTCACTAAAAATGAA  CrOMT2_Nta GGCAAGGTTGAGAGATTGTACGGCCTCGCTCCAGTTTGTAAATTTCTTACCAAAAATGAA  ***** ******** * ** ***** ** ** *********** ** ** *********  CrOMT2 GATGGTGTTACACTTTCCGATCTTTGTCTCATGAACCAAGACAAGGTTCTCATGGAGAGC  CrOMT2_Nta GATGGAGTGACGCTAAGTGATTTATGCTTGATGAACCAAGACAAAGTTCTCATGGAGAGC  ***** ** ** ** *** * ** * ************** ***************  CrOMT2 TGGTACTACTTAAAAGATGCAGTGCTTGAAGGTGGCATTCCATTTAACAAGGCCTATGGG  CrOMT2_Nta TGGTATTACCTGAAAGATGCTGTACTTGAGGGAGGCATACCATTTAACAAAGCTTATGGA  ***** *** * ******** ** ***** ** ***** *********** ** *****  CrOMT2 ATGAATGCATTCGATTACCATGGCAAAGATCTAAGATTCAACAAGATTTTCAACAATGGA  CrOMT2_Nta ATGAATGCATTTGATTATCATGGAAAAGACTTGAGGTTCAACAAGATATTTAATAATGGT  *********** ***** ***** ***** * ** *********** ** ** *****  CrOMT2 ATGTCTTCTCATTCTACCATTACCATGAAGAAAATTCTTGAAAATTACAAAGGGTTTGAA  CrOMT2_Nta ATGAGTTCTCACTCGACAATTACCATGAAGAAAATCTTAGAAAATTACAAGGGCTTTGAG  *** ****** ** ** ***************** * *********** ** *****  CrOMT2 GGCCTCAACTCAGTTGTCGACGTTGGTGGTGGAATTGGAGCCACACTTAACATGATTATC  CrOMT2_Nta GGGTTAAACTCTGTGGTCGATGTCGGTGGAGGTATTGGTGCAACTCTTAATATGATCATA  ** * ***** ** ***** ** ***** ** ***** ** ** ***** ***** **  CrOMT2 TCCAAGTATCCATCGATTAAAGGCATCAACTTTGATTTGCCACATGTTATTCAGGATGCT  CrOMT2_Nta TCCAAGTATCCATCAATCAAAGGAATAAATTTTGACCTTCCTCACGTGATTCAAGATGCT  ************** ** ***** ** ** ***** * ** ** ** ***** ******  CrOMT2 CCAGCTTTTCCTGGTGTCGAGCATGTTGGGGGAGACATGTTTGTTAGTGTTCCAAAGGGA  CrOMT2_Nta CCAGCCTTTCCTGGGGTAGAGCATGTAGGTGGAGATATGTTCGTGAGCGTTCCCAAAGGT  ***** ******** ** ******** ** ***** ***** ** ** ***** ** **  CrOMT2 GATGCCATTTTTATCAAGTGGATATGTCATGATTGGAGTGATGAGCACTGCGTGAAATTC  CrOMT2_Nta GATGCCATCTTCATAAAATGGATTTGTCATGATTGGTCTGATGAACATTGTGTTAAGTTT  ******** ** ** ** ***** ************ ****** ** ** ** ** **  CrOMT2 TTGAAGAACTGCTATGAAGCACTCCCAGTAAATGGGAAAGTCATTGTTGCTGAATCTATC  CrOMT2_Nta TTGAAGAATTGCTATGAAGCTTTGCCTGTCAATGGAAAGGTGATTGTTGCTGAAAGCATT  ******** *********** * ** ** ***** ** ** ************ **  CrOMT2 CTCCCAGTAACCCCGGACACAAGCCTCGCATCCAAAGTAGTCATCCATGTCGACTGCATC  CrOMT2_Nta CTGCCGGTTACTCCGGACACTAGTTTGGCATCAAAGGTTGTTATTCATGTTGATTGCATC  ** ** ** ** ******** ** * ***** ** ** ** ** ***** ** ******  CrOMT2 ATGTTGGCTCATAACCCGGGTGGCAAAGAGAGGACTGAACAAGAGTTCAGAGCATTGGCT  CrOMT2_Nta ATGCTTGCTCACAATCCTGGTGGTAAAGAAAGAACAGAGCAAGAATTTAGGGCTCTTGCC  *** * ***** ** ** ***** ***** ** ** ** ***** ** ** ** * **  CrOMT2 AAGGCTGCTGGATTCCAAGGTTTCCAAGTTGTGAGCTCTGCTTTTAATACTTACATTATG  CrOMT2_Nta AAGGCTGCTGGGTTTCAGGGTTTCCAGGTAGTGTCGTCAGCTTTCAATACATATATTATG  *********** ** ** ******** ** *** ** ***** ***** ** ******  CrOMT2 GAATTTCTCAAGAGTGCTTGA  CrOMT2_Nta GAATTCTTGAAAAGTGCGTGA  ***** * ** ***** *** |
| CsOMT21  (77%) | CsOMT21 ATGGGTTCAACAGGAATAGAGACCCAAATGACCCCAACCCAAATATCCGACGAAGAAGCC  CsOMT21_Nta ATGGGATCTACTGGAATTGAAACTCAAATGACTCCGACCCAGATTTCTGATGAGGAGGCA  ***** ** ** ***** ** ** ******** ** ***** ** ** ** ** ** **  CsOMT21 AACCTCTTCGCCATGCAATTAGCCAGTGCCTCAGTCTTACCCATGGTTCTCAAAGCAGCT  CsOMT21_Nta AACCTCTTTGCAATGCAGTTGGCTAGTGCATCAGTCCTACCAATGGTTTTGAAGGCTGCT  ******** ** ***** ** ** ***** ****** **** ****** * ** ** ***  CsOMT21 TTAGAGCTCGACCTCTTGGAGATCATAGCCAAGGCCGGTCCAGGCGCGTTTCTCTCACCT  CsOMT21_Nta TTAGAGTTGGACCTTCTTGAAATCATAGCAAAAGCAGGACCTGGTGCATTTCTTTCACCC  ****** * ***** * ** ******** ** ** ** ** ** ** ***** *****  CsOMT21 TCCGACATAGCTCAACAGCTTCCGACTCAGAACCCAGACGCCCCGGTGATGCTGGACCGG  CsOMT21_Nta AGTGACATTGCTCAACAACTGCCCACTCAGAATCCAGATGCCCCTGTCATGCTGGACAGA  ***** ******** ** ** ******** ***** ***** ** ********* *  CsOMT21 ATGCTGAGACTGTTGGCTAGCTACAACGTGGTGACGTACTCGCTGCGTGAGCGTGAGACG  CsOMT21_Nta ATGCTTCGCCTGCTAGCCAGTTACAATGTTGTTACTTATTCTTTGCGAGAGAGGGAAACT  ***** * *** * ** ** ***** ** ** ** ** ** **** *** * ** **  CsOMT21 GCGGAAGAGGAAGGGAAGGTGGAGAGGCTTTATGGGTTGGCTCCGGTGAGTAAATATCTG  CsOMT21_Nta GCTGAGGAAGAAGGGAAGGTTGAGAGACTTTATGGATTGGCACCAGTGAGCAAATACCTC  ** ** ** *********** ***** ******** ***** ** ***** ***** **  CsOMT21 ACGAAGAATGAAGATGGAGTCTCCATTGCTCCTCTTTGTCTCATGAACCAGGATAAGGTT  CsOMT21_Nta ACCAAAAATGAAGATGGAGTATCCATTGCTCCGCTGTGTTTGATGAATCAAGATAAGGTG  ** ** ************** *********** ** *** * ***** ** ********  CsOMT21 CTTATGGAGAGTTGGTATCACTTAAAAGATGCAGTACTTGATGGAGGAATACCTTTCAAC  CsOMT21_Nta CTTATGGAATCTTGGTACCATCTCAAAGATGCTGTGTTAGATGGCGGTATACCATTTAAC  ******** ****** ** * ******** ** * ***** ** ***** ** ***  CsOMT21 AAGGCATATGGAATGACAGCATTTGAATATCATGGAACCGATCAAAGGTTCAATAAAATC  CsOMT21_Nta AAGGCATATGGCATGACAGCGTTTGAATATCACGGCACTGATCAAAGGTTCAACAAAATT  *********** ******** *********** ** ** ************** *****  CsOMT21 TTTAATAGAGGAATGTCCGACCACTCGACTATTACCATGAAAAAAATCCTCGAAACTTAC  CsOMT21_Nta TTTAACCGTGGAATGTCGGATCATTCCACAATAACAATGAAGAAAATCTTGGAAACATAT  ***** * ******** ** ** ** ** ** ** ***** ****** * ***** **  CsOMT21 AAGGGTTTCGAGGGTCTTAACTCGATTGTTGATGTTGGTGGTGGTACTGGAGCTGTTGTT  CsOMT21_Nta AAGGGATTTGAGGGTTTGAATTCAATTGTTGATGTTGGTGGTGGAACAGGTGCTGTTGTA  ***** ** ****** * ** ** ******************** ** ** ********  CsOMT21 AACATGATCGTCTCTAAGTACCCTACTATTAAGGGTATTAACTTCGATTTGCCTCATGTC  CsOMT21_Nta AACATGATTGTCAGCAAGTATCCAACAATAAAAGGAATAAATTTTGATTTGCCTCATGTC  ******** *** ***** ** ** ** ** ** ** ** ** ***************  CsOMT21 ATCGAAGATGCACCTCCATTGACCGGTGTAGAGCATGTTGGAGGAGACATGTTTGTAAGT  CsOMT21_Nta ATTGAGGACGCCCCTCCTTTAACAGGTGTTGAACACGTGGGGGGAGACATGTTTGTTTCG  ** ** ** ** ***** ** ** ***** ** ** ** ** **************  CsOMT21 GTACCAAAAGGAGATGCAATTTTCATGAAGTGGATTTGCCATGATTGGAGCGATGAACAC  CsOMT21_Nta GTGCCAAAGGGTGATGCGATCTTCATGAAATGGATATGCCATGATTGGAGTGATGAACAT  ** ***** ** ***** ** ******** ***** ************** ********  CsOMT21 TGCTTGAAATTCTTGAAGAACTGCCACGCTGCACTGCCCGAACACGGAAAAGTGATCGTG  CsOMT21_Nta TGTTTAAAGTTTCTGAAGAATTGTCATGCTGCACTTCCTGAGCACGGGAAAGTAATTGTA  ** ** ** ** ******* ** ** ******** ** ** ***** ***** ** **  CsOMT21 GCGGAGTGCATTCTTCCGGTGGCACCGGACTCGAGCCTTGCCACAAAGAGTACGGTCCAC  CsOMT21_Nta GCTGAATGTATCCTTCCAGTTGCACCTGACTCTTCTCTAGCAACAAAGTCAACGGTACAC  ** ** ** ** ***** ** ***** ***** ** ** ****** ***** ***  CsOMT21 ATTGATGTGATCATGTTGGCCCATAACCCTGGTGGCAAAGAGAGAACAGAGAAAGAGTTT  CsOMT21_Nta ATTGATGTGATCATGCTAGCTCATAATCCTGGAGGTAAAGAAAGAACCGAAAAGGAATTC  *************** * ** ***** ***** ** ***** ***** ** ** ** **  CsOMT21 GAGGCATTGGCTAAGGGAGCTGGCTTTAAAGGCTTCAAAGTCCATTGCAATGCTTTCAAT  CsOMT21_Nta GAAGCTTTAGCCAAAGGCGCGGGGTTCAAAGGTTTCAAGGTTCATTGCAATGCTTTCAAT  ** ** ** ** ** ** ** ** ** ***** ***** ** ******************  CsOMT21 ACCCATATCATGGAATTTCTCAAGACCATTTAA  CsOMT21_Nta ACTCATATTATGGAGTTTTTAAAAACTATATGA  ** ***** ***** *** * ** ** ** * * |

**Supplementary Table 2.** List of primers used in this study.

| Purpose | Oligo Name | Oligo Sequences |
| --- | --- | --- |
| Construction of Level 0 modules and sequence confirmation of Level M vector | Lev0seq_F | CCTGTCGGGTTTCGCCACCT |
|  | Lev0seq_R | GCCGTTACCACCGCTGCGTT |
|  | Levm1seq_F | GCCACCTGACGTCTAAGAAACC |
|  | Levm1seq_R | AGCGAGGAAGCGGAAGAGCG |
|  | RB_short_F1 | GCATGCACATACAAATGGACG |
|  | LB_R1 | GGCTGGTGGCAGGATATATTG |
|  | CsOMT21-CDS1-F | TTGAAGACAAAATGGGATCTACTGGAATTG |
|  | CsOMT21-CDS1-R | TTGAAGACAAAAGCTCATATAGTTTTTAAAAAC |
|  | CrOMT2_Nta_R | TTGAAGACAAAAGCTCACGCAC |
|  | CrOMT2_Nta_F | TTGAAGACAAAATGGGAAGCAC |
|  | ROMT9_Nta_R | TTGAAGACAAAAGCTCATTTGG |
|  | ROMT9_Nta_F | TTGAAGACAAAATGGTGGATAG |
|  | OsF3'H_Nta_R | TTGAAGACAAAAGCTCAAACACC |
|  | OsF3'H_Nta_F | TTGAAGACAAAATGGACGTAGTGC |
|  | ZmFNSI_Nta_R | TTGAAGACAAAAGCTCAGGTACG |
|  | ZmFNSI_Nta_F | TTGAAGACAAAATGGCCGAGCATC |
|  | OsFNS_Nta_R | TTGAAGACAAAAGCTCACAGGAG |
|  | OsFNS_Nta_F | TTGAAGACAAAATGGCATCATTG |
|  | OsCHS_Nta_R | TTGAAGACAAAAGCTCATGCCGCG |
|  | OsCHS_Nta_F | TTGAAGACAAAATGGCTGCTGCAG |
|  | CaMV35Spro-F2 | GAGGTCAACATGGTGGAGCAC |
|  | TerAtuOCS-seqF | GAAGTGCAGGTCAAACCTTGAC |
|  | AtPAL-RT-R | CTGTTCGGGATAGCCGATGTTCCG |
|  | AtPAL-RT-F | CGCGGATGATCCTTGTAGCGCAAC |
|  | CaMV35S-Ter-seqF | GGGTTTCGCTCATGTGTTGAGC |
|  | Pro-AtRbcS2B-seqF | CCGATATCTACAATTTGACGCC |
|  | OsF3'H-RT-F | GTAGGATGCATGCTGATGTCGACG |
| Gene expression analysis | PP2A-RT-F | GACCCTGATGTTGATGTTCGCT |
|  | PP2A-RT-R | GAGGGATTTGAAGAGAGATTTC |
|  | CsOMT21-RT-F | CGGTGCCAAAGGGTGATGCGAT |
|  | CsOMT21-RT-R | GAGAAGAGTCAGGTGCAACTGG |
|  | CrOMT2-RT-R2 | TTGAAAGCTGACGACACTACCTGG |
|  | CrOMT2-RT-F2 | GCATCATGCTTGCTCACAATCCTG |
|  | ROMT9-RT-R | CCAGTGAATCCAGCTGCTCTTGC |
|  | ROMT9-RT-F | GCTATGATGCCTTGCCTGAGCACG |
|  | OsF3'H-RT-R2 | GTCTAATCAACTCCGCTAGTGCCC |
|  | OsF3'H-RT-F2 | GAAGAGCAGAAGTTGGACGGTGAC |
|  | ZmFNSI-RT-R | CAGCCGGAGTATCCTCTGTGACAAG |
|  | ZmFNSI-RT-F | GGTCCTTCATGCTGGGCAATGGGT |
|  | OsFNS-RT-R2 | CCTAGCACACACCAAACCATCTGC |
|  | OsFNS-RT-F2 | CATGCCATTTGGATCTGGAAGGAG |
|  | AtPAL-RT-R2 | GAACTCTTCTCCAGGCGACGTCAC |
|  | AtPAL-RT-F2 | AGCCTACGATAACGGAACATCGGC |
|  | OsCHS-RT-R2 | GCATGACCATCTTCTGCACTACG |
|  | OsCHS-RT-F2 | TTGAAGCTAAGGTCGGCCTTGAC |
| Molecular confirmation of transgenic plants | BAR_seqF | GCTTACGATTGGACTGCTGAG |
|  | BAR_R | TCAAATCTCAGTCACAGGAAGAAC |

**Supplementary Table 3.** DNA parts used for module assembly..

| No. | Modules | Promoters | CDS | Terminators | Level M  end-linker 6 | Acceptors |
| --- | --- | --- | --- | --- | --- | --- |
| 1 | Level 0 |  | AtPAL |  |  | pAGM1287 |
| 2 |  |  | OsCHS ,OsFNS, ZmFNSI, OsF3'H, CsOMT21, ROMT9, or CrOMT2 |  |  | pICH41308 |
| 3 | AtPAL level 1 | Pro+5U, GGAG-ProG10-90-AATG (pTEI038GB) | AtPAL | GCTT_3U+Ter-CaMV35S (pICH41414) |  | pICH47751 |
| 4 | OsCHS level 1 | Pro+5U, GGAG-ProAtUBQ10-AATG (pJOG684*GB) | OsCHS | GCTT_3U+Ter -Atug7 (pICH72400) |  | pICH47761 |
| 5 | OsFNS level 1 | GGAG_Pro-AtRbcS2B+5U-Pro-AtRbcS2B_AATG (pICH45195) | OsFNS | GCTT_3U+Ter -AtuNos (pICH41421) |  | pICH47772 |
| 6 | ZmFNSI level 1 | GGAG_Pro-AtRbcS2B+5U-Pro-AtRbcS2B_AATG (pICH45195) | ZmFNSI | GCTT_3U+Ter -AtuNos (pICH41421) |  | pICH47772 |
| 7 | OsF3'H level 1 | GGAG_Pro- AtLHB1B1+5U-Pro-AtLHB1B1 _AATG (pICH45214) | OsF3'H | GCTT_3U+Ter -AtuMas (pICH77901) |  | pICH47781 |
| 8 | ROMT9 level 1 | GGAG_Pro-CaMV35SDouble_5UTMV_AATG (pICH51288) | ROMT9 | GCTT_3U+Ter -AtuOcs (pICH41432) |  | pICH47742 |
| 9 | CrOMT2 level 1 | GGAG_Pro-CaMV35SDouble_5UTMV_AATG (pICH51288) | CrOMT2 | GCTT_3U+Ter -AtuOcs (pICH41432) |  | pICH47742 |
| 10 | CsOMT21 level 1 | GGAG_Pro-CaMV35SDouble_5UTMV_AATG (pICH51288) | CsOMT21 | GCTT_3U+Ter -AtuOcs (pICH41432) |  | pICH47742 |
| 11 | BAR level 1 | GGAG_Pro-AtuNos_5U-AtuNos_CDBAR_Ter-AtuNos_CGCT (pICSL70005) | | |  | pICH47732 |
| 12 | PCFF'O Lv. M | Each level 1 modules (AtPAL, OsCHS ,OsFNS, OsF3'H, CrOMT2) | | | pICH50927 | pAGM8043 |
| 13 | BAR+PCFF'O Lv. M | Each level 1 modules (BAR, AtPAL, OsCHS ,OsFNS, OsF3'H, CrOMT2) | | | pICH50927 | pAGM8031 |
